# Progressive drought alters architectural and anatomical traits of rice roots

**DOI:** 10.1186/s12284-018-0252-z

**Published:** 2018-12-04

**Authors:** Mohamed Hazman, Kathleen M. Brown

**Affiliations:** 1 Department of Plant Science, The Pennsylvania State University, 102 Tyson Building, University Park, University Park, PA 16802-4200 USA; 2 Agricultural Genetic Engineering Research Institute (AGERI), Agricultural Research Centre (ARC), 9 Gamma St., Giza, 12619 Egypt

**Keywords:** Rice roots, Drought, Laser ablation tomography images, Architecture, Anatomy, Lignin

## Abstract

**Background:**

Root architectural and anatomical phenotypes are important for adaptation to drought. Many rice-growing regions face increasing water scarcity. This study describes drought responses of 11 Egyptian rice cultivars with emphasis on plastic root responses that may enhance drought adaptation.

**Results:**

Eleven Egyptian rice cultivars were phenotyped for root architectural and anatomical traits after 6 weeks growth in soil mesocosms under well-watered conditions. Four of these cultivars were more intensively phenotyped under progressive drought stress in mesocosms, using a system where more moisture was available at depth than near the surface. In response to drought stress, all cultivars significantly reduced nodal root number while increasing large lateral root branching density and total lateral root length in the deepest portions of the mesocosm, where moisture was available. Nodal root cross-sectional area, but not stele area, was reduced by drought stress, especially in the basal segments of the root, and the number of late metaxylem vessels was reduced in only one cultivar. Alterations in deposition of lignin were detected by UV illumination from laser ablation tomography, enhanced by digital staining, and confirmed with standard histochemical methods. In well-watered plants, the sclerenchyma and endodermis were heavily lignified, and lignin was also visible throughout the epidermis and cortex. Under drought stress, very little lignin was detected in the outer cell layers and none in the cortex of nodal roots, but lignin deposition was enhanced in the stele. Root anatomical phenes, including cross-section area and metaxylem vessel number and lignin deposition varied dramatically along large lateral root axes under drought stress, with increasing diameter and less lignification of the stele in successive samples taken from the base to the root apex.

**Conclusions:**

Root architectural and anatomical traits varied significantly among a set of Egyptian cultivars. Most traits were plastic, i.e. changed significantly with drought treatment, and, in many cases, plasticity was cultivar-dependent. These phenotypic alterations may function to enhance water uptake efficiency. Increased large lateral root branching in the deep soil should maintain water acquisition, while water transport during drought should be secured with a more extensively lignified stele.

**Electronic supplementary material:**

The online version of this article (10.1186/s12284-018-0252-z) contains supplementary material, which is available to authorized users.

## Background

Drought avoidance is one of the most important strategies for maintaining crop yields in water-limited environments. Drought avoidance is most often attributed to root phenes that support better water capture and transport to the shoot (Clark et al. 2002; Lynch et al., 2014). Investigating the architectural and anatomical phenes that contribute to rooting depth is essential for improving crop performance under drought stress (Lynch 2014). Compared to vegetative growth and yield, root traits have not been popular breeding objectives, partly due to the labor-intensive nature of root system phenotyping under agronomically relevant conditions. The recent development of high-throughput phenotyping platforms has increased the potential for associating root phenes with water acquisition from drying soil in cereals, including rice (Henry et al. 2012; Kadam et al. 2017) and maize (Lynch et al., 2014; Lynch 2014; Lynch 2018).

Production of rice consumes large amounts of water, two to three times more than dry-land cereals. Attempts to reduce water usage in rice, a semi-aquatic crop, have been limited by its sensitivity to drought stress (Wassmann et al. 2009). Since roots are responsible for water acquisition, root architectural and anatomical traits are key to breeding strategies aimed at drought avoidance. Root traits associated with improved drought avoidance include steeper nodal root angle to increase root depth, and larger diameter roots, which are associated with greater ability to penetrate hard soils to access deep water (Gowda et al. 2011).

Rice possesses a fibrous root system comprised of a mix of embryonic and post-embryonic roots with multiple branching orders (Rebouillat et al. 2009). Post-embryonic roots consist of nodal roots arising from each tiller and numerous lateral roots branching from these axes. There are two types of lateral roots in rice, small lateral roots and large lateral roots. Small lateral roots are shorter, more abundant, ageotropic and do not ramify, while large lateral roots are much longer, less abundant, geotropically positive and highly branched. Root length density in deeper soil layers is highly correlated with soil exploration and water uptake efficiency (Kamoshita et al. 2000; Siopongco et al. 2005). Since rice root architecture is characterized by relatively short nodal roots (shorter than maize or barley), the large lateral roots are likely to contribute substantially to deep soil exploration. Increased lateral root formation under drought stress was suggested as a potentially useful adaptation to drought in lowland rice (Morita et al. 2002; Henry et al. 2012). Several studies have associated improved shoot biomass, water uptake, and photosynthesis under drought with plasticity in lateral root development (Suralta et al. 2010; Kano et al. 2011; Kano-Nakata et al. 2011).

Root anatomical phenes influence radial and axial water transport in roots, which would be expected to influence the efficiency of water uptake and distribution (Lynch et al., 2014). Xylem vessel traits (number, diameter and area) affect axial water conductance while cortical traits and the presence of suberized cell layers may affect radial conductance. Larger xylem vessels and thicker roots are characteristic of upland rice and associated with improved drought tolerance (Gowda et al. 2011).

Like many rice-growing areas, Egypt is faced with increasingly limited irrigation water to support rice production. Egypt is the largest rice-producing country in the West Asia/ North Africa region, producing about 4.8 million tons of paddy rice in 2016 (U.S. Department of Agriculture FAS 2018). Rice production in Egypt has always been constrained by the availability of irrigation water, since the region receives minimal rainfall. Production has declined in recent years and is predicted to decline dramatically this year due to government-imposed 33% reduction in allotted rice growing area for 2018 (Wally and Beillard 2018). These restrictions are motivated by declining water resources to support this popular but water-intensive crop. Sustainable rice production will require cultivars adapted to water-saving management strategies that do not involve continuous flooding, which is the currently most common practice. We hypothesize that cultivars with the ability to adapt their roots to reduced moisture by maintaining root depth and deep branching will be better suited to progressive drought scenarios. Therefore, we investigated the architectural and anatomical traits of several rice cultivars in well-watered conditions and in response to progressive drought in greenhouse mesocosms.

## Material and Methods

### Plant Materials, Growth Conditions and Drought Treatment

Eleven publicly available lowland rice cultivars (*Oryza sativa*) developed by the scientific agricultural community in Egypt were obtained from the U.S. National Plant Germplasm System (https://npgsweb.ars-grin.gov/gringlobal/search.aspx). These cultivars varied for days to flowering and represent a range of varietal groups and subpopulations (Additional file 1: Table S1). In the first experiment, all 11 rice cultivars were grown under well-watered conditions to examine root architectural and anatomical traits. In the second experiment, four cultivars with high and comparable vigor in our growth system, Egypt 1, Egypt 5, Nabatat Asmar, and Nahda, were chosen for drought stress (DS) experiments. The experiments were performed in a greenhouse located on the campus of Pennsylvania State University, University Park, PA (40°48’ N, 77°51’ W). The caryopses were dehusked and surface sterilized according to (Hazman et al. 2015) and pre-germinated for 1 week prior to transplanting three healthy seedlings (reduced to one after 3 days) into 1.2 × 0.15 m plastic mesocosms. The mesocosms were lined with transparent high-density polyethylene film to facilitate root system excavation and sampling, and filled with a mixture of 40% (*v*/v) medium size commercial grade sand, 45% vermiculite, 5% perlite, and 10% clay topsoil (Hagerstown silt loam, mesic typic Hapludalf) collected from the Russell E. Larson Agricultural Research Center in Rock Spring, Pennsylvania. The mixture was fertilized with 50 g per mesocosm of Osmocote Plus Fertilizer, which includes micronutrients (Scotts Miracle-Gro Company, Marysville, OH USA). For well-watered treatments, plants were irrigated two times per day with water via drip irrigation (80–100% field capacity), and in addition manually received 50 ml per mesocosm per week of Yoshida nutrient solution (Yoshida et al. 1971). Plants were grown to the V8 stage (4 weeks) then harvested. In a separate experiment, four cultivars with similar vigor, Egypt 1, Egypt 5, Nabatat Asmar, and Nahda were grown under well-watered and drought stress conditions. Drought stress treatment was initiated after 2 weeks of growth by allowing the surface to dry gradually for 4 weeks. Soil moisture was monitored digitally with TDR (Time Domain Reflectometer) probes inserted 25 and 100 cm from the top of the medium of representative mesocosms (Campbell Scientific Inc., Utah, USA). There were no significant differences in volumetric moisture content among cultivars, as measured from the upper or lower sampling positions at the beginning of the drought stress or at the end of the experiment (data not shown). Both well-watered and drought stressed plants were harvested after 6 weeks growth.

### Root and Shoot Growth Measurements

At harvest, tillers were counted and shoots removed for biomass determination. For root sampling, root systems were removed from the mesocosms, facilitated by the presence of a plastic liner. The maximum root depth was recorded in the intact root system, then the root system was washed with a hose to remove the medium. Nodal root number was recorded, and a representative nodal root was removed and stored in 70% ethanol for analysis of additional architectural and anatomical traits. The remainder of the root system and the harvested shoots were dried in an 80 °C oven for 72 h for dry biomass determinations. Percentage of dry biomass reduction was calculated according to the formula: (WW-DS/WW) × 100, where WW = well-watered and DS = drought stress.

### Root Architecture Measurements

Total length and branching density of large lateral roots (LLR) and small lateral roots (SLR) on the preserved nodal root sample were measured using WinRhizo (Regent Instruments, Quebec, Canada). The nodal root was divided into several lengths and each length was scanned individually using a flatbed scanner at a resolution of 600 dpi (HP ScanJet, Hewlett Packard, USA). WinRhizo was used to quantify lengths of small and large lateral roots according to the following diameter classes: less than 0.25 mm SLR, 0.25 to 0.60 mm for LLR, and larger than 0.60 mm for nodal roots. SLR were distinguished from LLR based on small diameter and lack of secondary branching. Branching density was calculated by number of tips relative to the length of the nodal root. These measurements were calculated separately for the apical 20 cm and for the remaining basal portion of the nodal root.

### Measurements of Root Anatomical Phenes

Dissected rice root segments were imaged using laser ablation tomography (LAT). Preserved root segments from nodal roots were collected at 20 cm from root base and 10 cm from root apex and dried with an automated critical point dryer (CPD, Leica EM CPD 300, Leica Microsystem, Vienna, Austria) according to the manufacturer protocol. For LLR anatomy, several LLR of one rice cultivar (Egypt 1) were gently dissected from the nodal root axis and divided with a razor blade into three parts for well-watered plants and five parts for drought-stressed plants and dried using CPD as previously described. Segments of dried nodal and LLR roots were ablated by a laser beam (Avia 7000, 355 nm pulsed laser) to vaporize the root at the camera focal plane ahead of an imaging stage, then cross-section images were taken using a Canon T3i camera with a 5x micro lens (MP-E 65 mm) on the laser-illuminated surface (Chimungu et al. 2015). The resulting images were analyzed by the software MIPAR (Sosa et al. 2014) to obtain areas of the cross-section, stele, and metaxylem vessels. The theoretical axial water conductance of nodal roots was calculated according to Tyree and Ewers (Tyree and Ewers 2018). Picasa software was used to convert the semi-monochromatic high-quality LAT images into complementary diadic colored images with a bright white background using the following recipe: a) Heat map the laser image to 0% hue and nearly 50% fade, b) invert the colors, c) cross process, then finally d) Orton-ish by 0% bloom, almost 50% brightness and 0% fade. This enabled us to qualitatively distinguish the secondary cell wall elements, and is hereafter referred to as “*digital staining*”.

### Histochemical Staining of Secondary Cell Wall Elements

For histochemistry, ethanol-preserved nodal root segments of one representative cultivar (Egypt 5) were washed gently with deionized water and hand sectioned using a razor blade. Thin sections were stained with three different lignin dyes: 0.02% toluidine blue, 3% phloroglucinol-HCL (Weisner staining) and 0.5% potassium permanganate (Mäule staining) (Sigma-Aldrich, USA). Images of the root sections were acquired with a Nikon SMZ 1500 stereoscope (Nikon, Japan) with 50× and 100× magnification (Pradhan Mitra and Loqué 2014).

### Experimental Design and Statistical Analysis

A randomized complete block design was with at least three independent biological replications. SPSS (IBM Statistics, USA) software was used for statistical tests including mean separations by Tukey’s Honestly Significant Difference (HSD) test, with a significance level of *P* ≤ 0.05, Pearson correlation coefficient, and ANOVA analysis.

## Results

### Root Morphology and Anatomy of 11 Egyptian Cultivars Under Well-watered Conditions

**Fig. 1:**
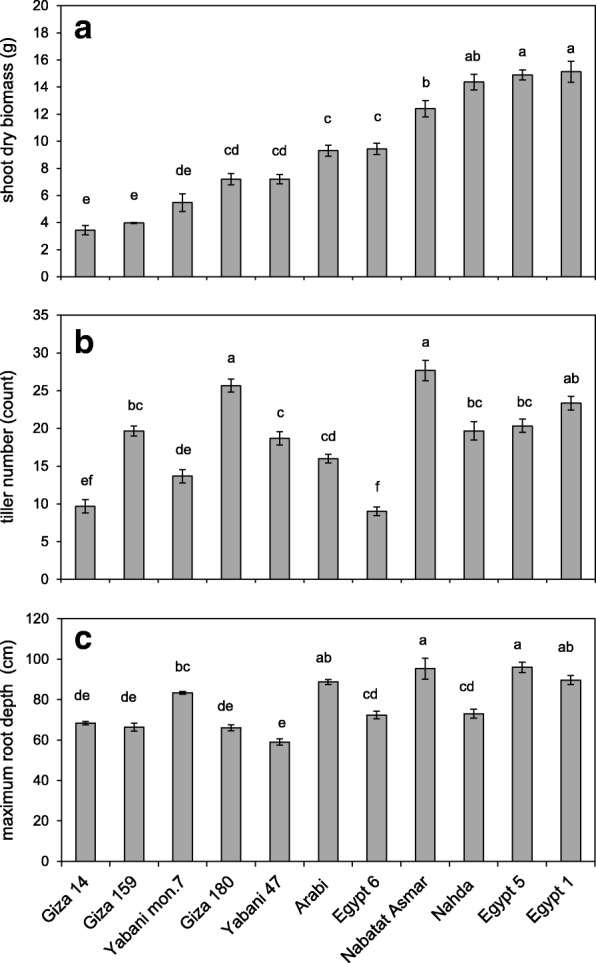
**a** Shoot dry biomass, **b** tiller number, and **c** maximum root depth of 11 Egyptian rice cultivars grown in the greenhouse under well-watered conditions. Values shown are means of three replications ± SE. Means with the same letter are not significantly different according to Tukey’s Honest Significant Differences (HSD) test (*P* ≤ 0.05)

**Fig. 2:**
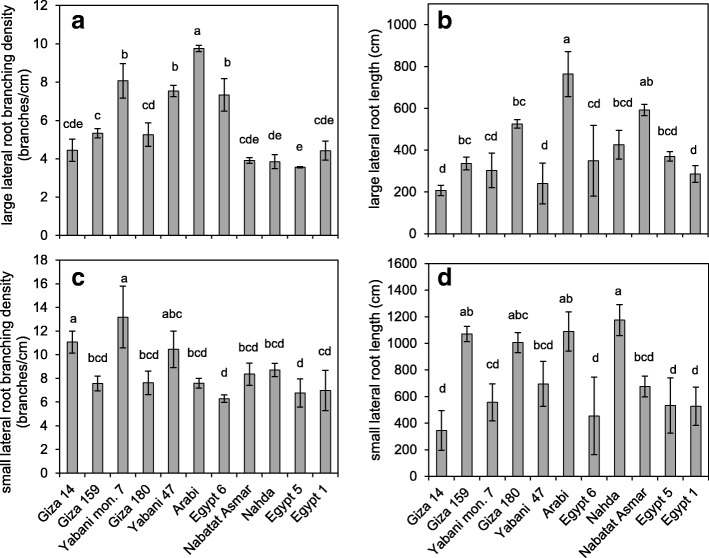
Genotypic variation among 11 Egyptian rice cultivars in **a** large lateral root branching density, **b** total large lateral root length per nodal root, **c** small lateral root branching density, and **d** total small lateral root length per nodal root in well-watered conditions. Values shown are means of three replications ± SE. Means with the same letter are not significantly different according to Tukey’s Honest Significant Differences (HSD) test (*P* ≤ 0.05)

**Fig. 3:**
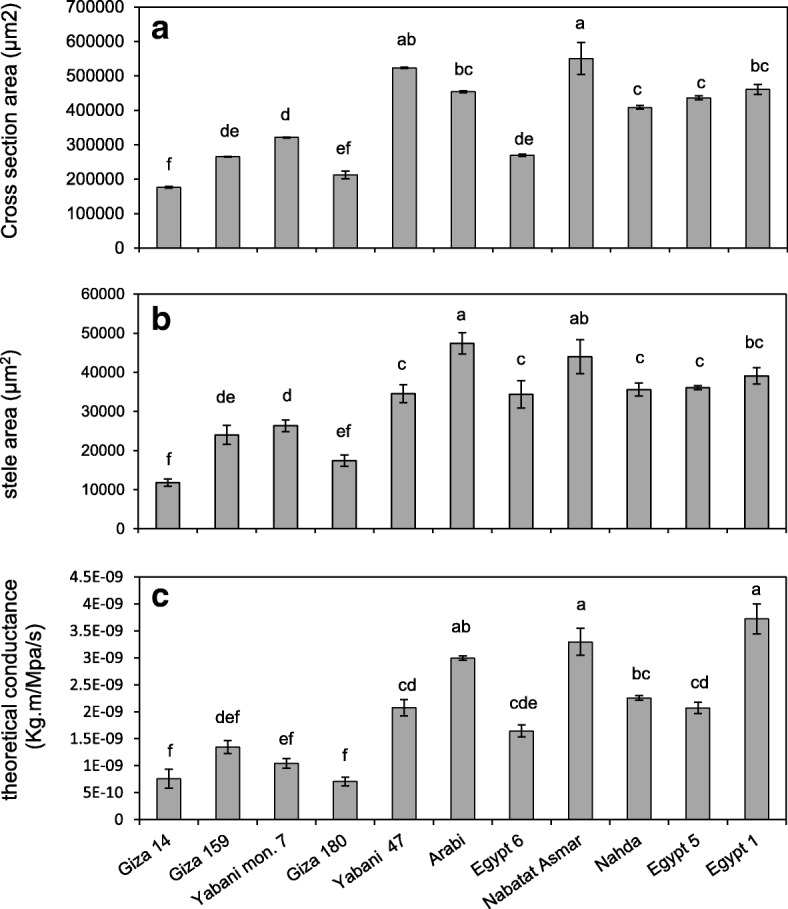
Nodal root anatomical phenes of 11 Egyptian rice cultivars grown in the greenhouse under well-watered conditions. **a** cross section area, **b** stele area, and **c** theoretical axial conductance. Values shown are means of three replications ± SE. Means with the same letter are not significantly different according to Tukey’s Honest Significant Differences (HSD) test (P ≤ 0.05)

We investigated root architectural and anatomical phenes of 11 cultivars of Egyptian paddy rice grown under well-watered, aerobic conditions (Additional file 1: Table S1). There was significant genetic variation in shoot dry biomass, tiller number and maximum root depth of 4-week-old plants (Fig. 1). Both root architectural (Fig. 2) and anatomical (Fig. 3) features showed significant genetic variation. Nahda, Egypt 1 and Egypt 5 produced the greatest shoot dry biomass. Tiller number was weakly correlated with shoot dry biomass and not correlated with maximum root depth (Fig. 1a, c and Table 1). Egypt 5 and Nabatat Asmar had the deepest roots while Yabani 47 had the shortest maximum root depth (Fig. 1c).

**Table 1:** Pearson’s correlations among root and shoot traits of 11 Egyptian rice cultivars under well-watered conditions

	Max. root depth	Large LRBD	Small LRBD	Large LRL	Small LRL	Cross section area	Stele area	Median MV area	Total MV area	MV No.	Theor. cond.	Shoot dry biomass	Tiller No.
Max. root depth	1												
Large LRBD	−0.135	1											
Small LRBD	−0.187	0.192	1										
Large LRL	0.245	0.208	−0.276	1									
Small LRL	−0.183	0.063	−0.111	0.733^**^	1								
Cross section area	0.484^**^	0.017	−0.081	0.173	0.068	1							
Stele area	0.414^*^	−0.271	−.538^**^	0.275	0.205	0.563^**^	1						
Median MV area	0.224	−0.008	0.303	0.145	0.259	0.342	0.267	1					
Total MV area	0.548^**^	0.020	−0.364^*^	0.346^*^	0.170	0.839^**^	0.630^**^	0.080	1				
MV No.	−0.398^*^	0.279	0.201	0.349^*^	0.504^**^	−0.199	−0.003	0.237	−0.280	1			
Theor. cond.	0.023	0.109	0.395^*^	0.207	0.337	0.137	0.191	0.871^**^	−0.120	0.631^**^	1		
Shoot dry biomass	0.624^**^	−0.399^*^	−0.475^**^	0.275	0.034	0.616^**^	0.568^**^	0.006	0.752^**^	−0.270	−0.161	1	
Tiller No.	0.259	−0.436^*^	−0.302	0.200	0.339	0.456^**^	0.664^**^	0.502^**^	0.378^*^	−0.016	0.372^*^	0.451^**^	1

*LRBD* Lateral root branching density, *LRL* Lateral root length, *MV* Metaxylem vessel, *Theor. cond* Theoretical conductance, *Max* Maximum, and *No.* Number significance indicated by * *p* ≤ 0.05; ** *p* ≤ 0.01

Root architecture phenes varied significantly among the rice cultivars (Fig. 2). Arabi had the greatest large lateral root branching density and length. Large and small lateral root branching densities were negatively correlated with shoot dry biomass and large lateral root branching density was negatively correlated with tiller number (Table 1). Small and large lateral root length were highly correlated with each other but were not correlated with lateral root branching density or with shoot biomass (Table 1).

Root anatomy also varied among cultivars (Fig. 3, Additional file 2: Figure S1). Nodal root cross-sectional area and stele area were positively correlated with shoot dry biomass, tiller number, maximum root depth, and each other (Table 1). Nodal root stele area was negatively correlated with small lateral root branching density and positively correlated with maximum root depth. While median metaxylem vessel area was positively correlated with tiller number, it was not correlated with shoot biomass or metaxylem vessel number. Theoretical axial water conductance, calculated from vessel size and number, was positively correlated with tiller number but not shoot biomass (Table 1).

**Fig. 4:**
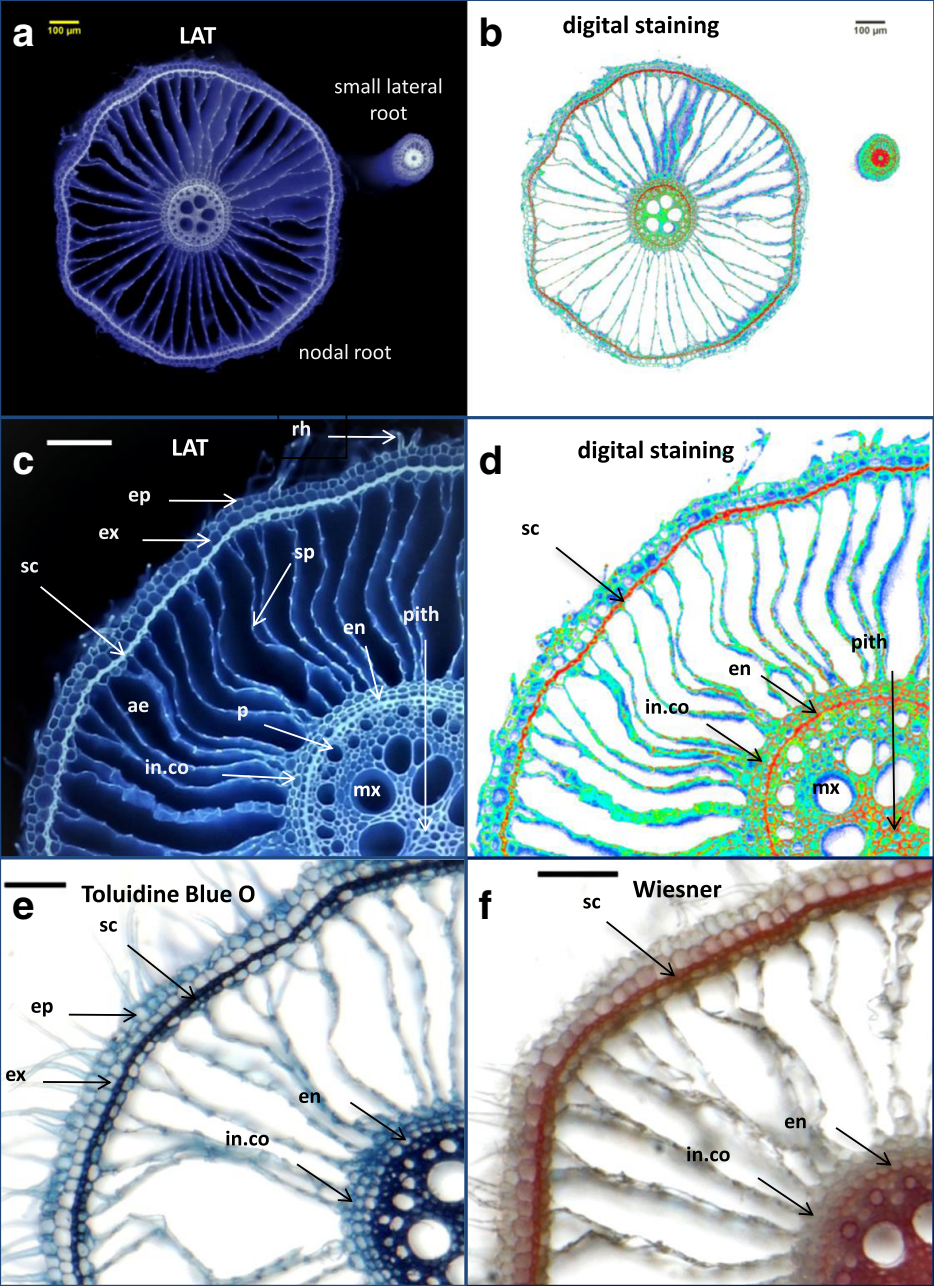
Comparison of Laser Ablation Tomography (LAT) images plus digital processing with standard histological methods for lignin assessment. Nodal root samples were collected from 4-week-old well-watered Arabi. **a** LAT image showing a nodal root and a small lateral root; **b** the same image digitally “stained”; **c** enlarged view of a nodal root cross section showing detailed anatomical structures including root hairs (rh), epidermis (ep), exodermis (ex), sclerenchyma (sc), aerenchyma (ae), septa (sp), inner cortical cells (in.co), endodermis (en), phloem (p), metaxylem vessel (mx), and pith; **d** digital staining of image “**c**” showing lignin deposition in cell walls of sc, in.co, en and pith. **e** Toluidine Blue O staining of hand cross sections of fresh nodal roots showing lignin deposition (blue color) in cells of sc, in co, and en, and **f** Wiesner or phloroglucinol-HCl staining showing lignin deposition (reddish-brown color) in sc and en. Horizontal scale bars represent 100 μm

In order to take the advantage of the variation in auto-fluorescence created by UV-laser beam excitation for lignin visualization, we developed simple approach to convert high quality laser ablation tomography (LAT) images of rice roots (Fig. 4a) into multichromatic images reflecting pixel intensities. These *digitally stained* images permit high-resolution assessment of tissue-level distribution of lignin in the sclerenchyma, endodermis and stele tissues (Fig. 4a-d). Comparison of these digitally stained images with conventional histochemical staining of lignin using toluidine blue O and Wiesner (phloroglucinol-HCl) (Fig. 4e-f) demonstrates the coincident indications of lignification. To show that this method is useful for other species, lignin was also digitally visualized in sweet corn primary roots (Additional file 3: Figure S2), where lignin deposition around outer cortical cells and stele cells was clearly visualized using digital staining of a high-quality LAT image.

### Drought Responses of Four Egyptian Cultivars: Shoot Growth and Root Architecture

**Fig. 5:**
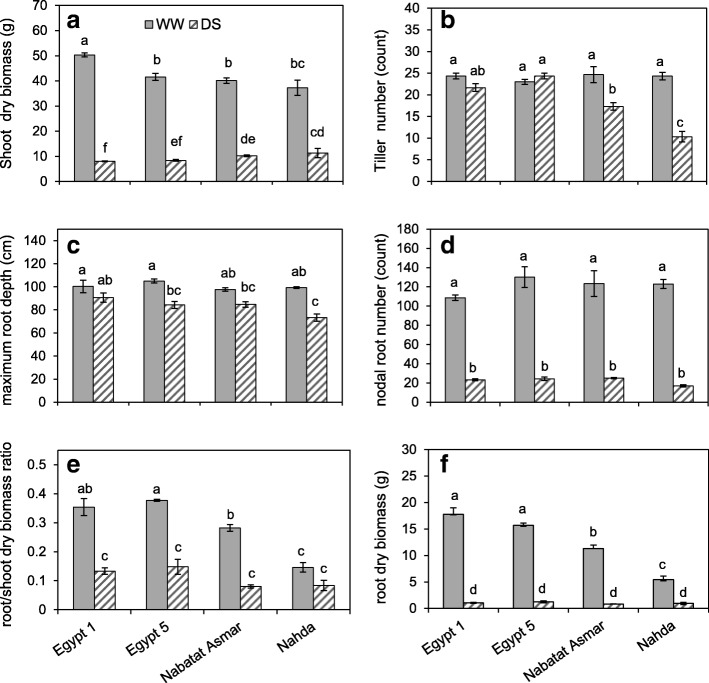
Effects of drought stress on **a** shoot dry biomass, **b** tiller number, **c** maximum root depth, **d** nodal root number, **e** root to shoot ratio, and **f** root dry biomass of cultivars Egypt 1, Egypt 5, Nabatat Asmar and Nahda. Values shown are means of three replications ± SE. Means with the same letter are not significantly different according to Tukey’s Honest Significant Differences (HSD) test (*P* ≤ 0.05)

Four cultivars with the greatest shoot dry biomass without drought (Egypt 1, Egypt 5, Nabatat Asmar and Nahda, Fig. 1) were selected for investigation of the effect of drought on rice root architecture and anatomy. Drought stress was imposed by stopping irrigation so that the upper part of the growth medium became gradually dry while the deep medium retained some moisture. Time domain reflectometry (TDR) probes showed that the volumetric water content (θ_*v*_) of droughted mesocosms was less than that of well-watered mesocosms, and drier at the top than at the bottom (Additional file 4: Figure S3). Drought stress significantly reduced shoot and root dry biomass and tiller number (Fig. 5, Table 2). Shoot biomass reduction ranged from 70% in Nahda to 84% in Egypt 1, and root biomass reduction ranged from 82.65% in Nahda to 92.81% in Nabatat Asmar (Fig. 5). Tiller number was significantly reduced in Nahda (58%) and Nabatat Asmar (30%).

**Table 2:** Analysis of variance for effects of cultivar and drought treatment on shoot and root traits

	Cultivar	Treatment	Cultivar x Treatment
Growth traits			
Shoot dry biomass	0.99	265.02**	23.35**
Tiller number	15.35**	60.84**	20.56**
Root dry biomass	44.80**	770.00**	41.40**
Root/shoot dry biomass ratio	29.51**	209.95**	10.11**
Root architectural traits			
Maximum root depth	1.16	474.88**	1.14
Nodal root number	3.69*	63.78**	2.89
Basal large lateral root branching density	0.20	12.68**	0.46
Basal small lateral root branching density	15**	10.11**	18.38**
Apical large lateral root branching density	2.66	201.42**	3.74*
Apical small lateral root branching density	6.59**	3.053	6.138**
Apical large lateral root length	1.78	40.28**	1.22
Apical small lateral root length	11.76**	74.06**	12.05**
Root anatomical traits			
Basal cross section area	10.02**	83.38**	5.84**
Basal stele area	2.31	0.16	6.28**
Ratio of basal cross-section to stele areas	12.46**	491.60**	10.85**
Basal median metaxylem vessel area	4.38*	0.29	1.33
Basal metaxylem vessel number	5.83**	12.50**	16.50**
Apical cross section area	10.02**	83.38**	5.84**
Apical stele area	3.09	8.08	0.97
Ratio of apical cross section to stele area	21.47**	322.40**	10.92**
Apical median metaxylem vessel area	1.88	5.67*	0.10
Apical metaxylem vessel number	5.86**	7.00*	3.57*

Values shown are *F* values, and *P* values are indicated by * *p*˂0.05 and ** *p*˂0.01

Maximum root depth did not vary among cultivars in well-watered plants but declined with drought by 20% to 26% (Fig. 5, Table 2). Drought stress reduced nodal root number to a much greater extent, averaging more than 80% reduction in all cultivars (Fig. 5). Drought stress significantly reduced root to shoot ratio in all cultivars except Nahda.

**Fig. 6:**
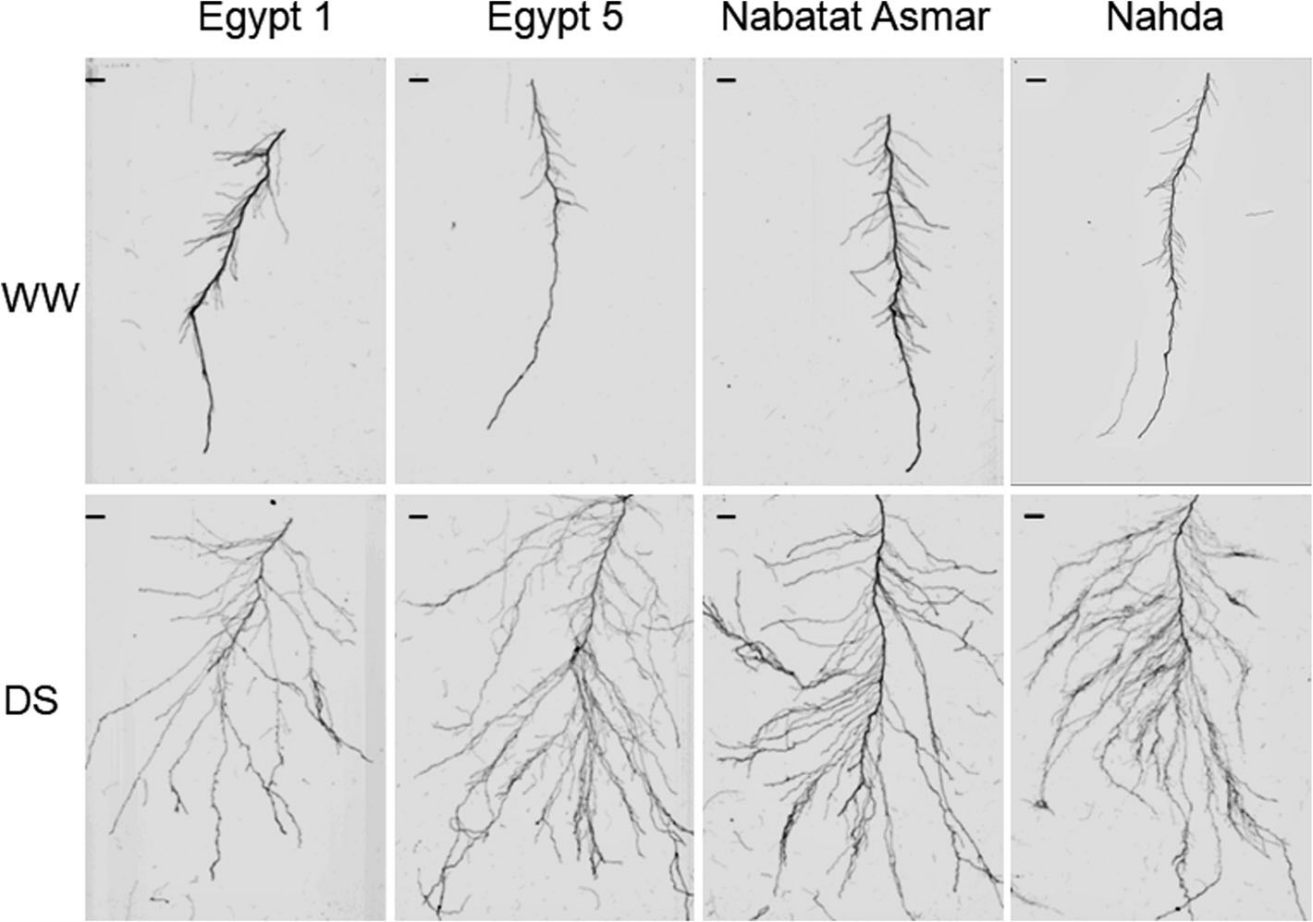
Representative images of the apical 20 cm of nodal roots from well-watered (WW) and drought stressed (DS) plants. Scale bar represents 10 mm

**Fig. 7:**
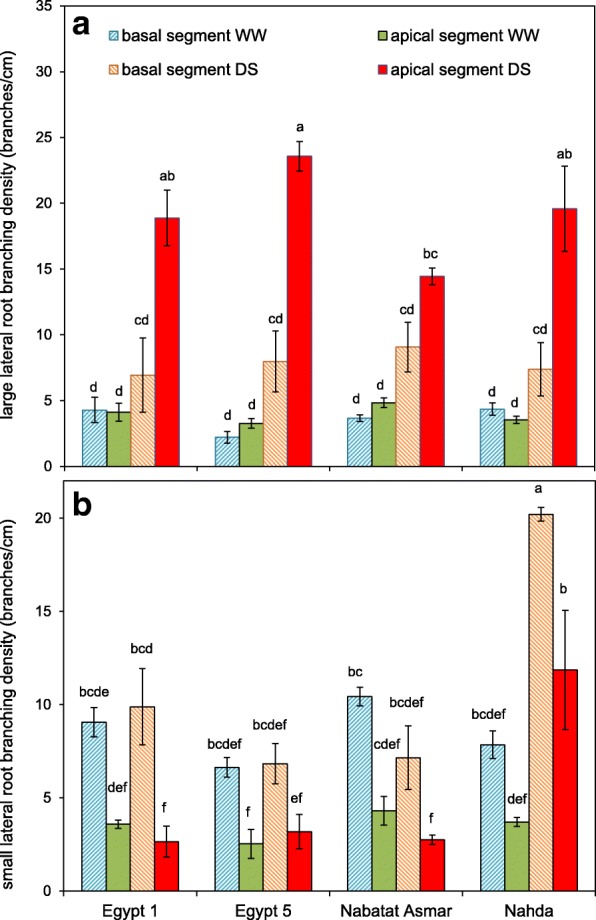
Lateral root branching density basal and apical segments of well-watered (WW) and drought stressed (DS) nodal roots of four rice cultivars. The basal segment consisted of the entire nodal root except for the apical 20 cm segment, which was evaluated separately. **a l**arge lateral root branching density and **b s**mall lateral root branching density. Values shown are means of three replications ± SE. Means with the same letter are not significantly different according to Tukey’s Honest Significant Differences (HSD) test (*P* ≤ 0.05)

Lateral root branching varied along nodal root axes, with greater branching in the deeper segments of the roots under drought (Fig. 6). We therefore separately evaluated lateral root branching in apical (deepest 20 cm) and basal (the rest of the root) nodal root segments (Fig. 7). Drought stress significantly increased large lateral root branching density especially in apical segments, and there was a significant cultivar x treatment interaction (Fig. 7, Table 2). The drought-induced increase in large lateral root branching density of the apical segment of nodal roots ranged from 3.0 (Nabatat Asmar) to 7.2-fold (Egypt 5). There was significant cultivar x treatment interaction for small lateral root branching density, but in this case the greater effect was on the basal segment (Table 2). Basal segments had greater small lateral root branching density than apical segments in all four cultivars (Fig. 7). Only Nahda had a significant increase in small lateral root branching density under drought stress, in both apical (3.2-fold) and basal segments (2.6-fold).

**Fig. 8:**
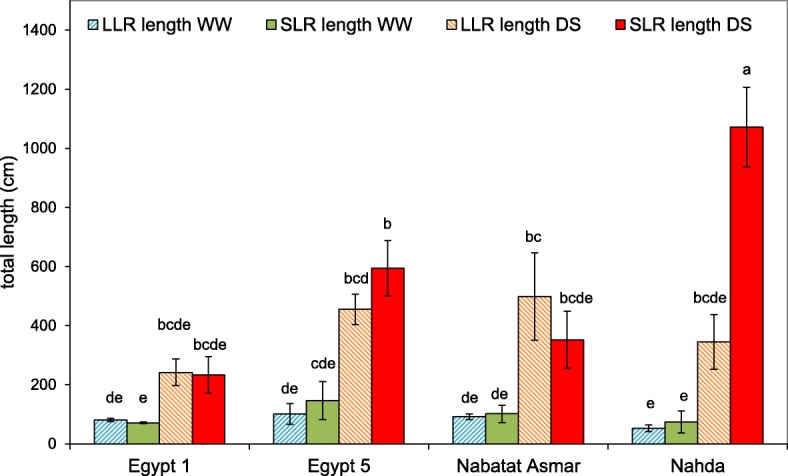
Effect of drought stress on large and small lateral root lengths in four rice cultivars. Root lengths are for the apical (deepest) 20 cm nodal root segment. Values shown are means of three replications ± SE. Means with the same letter are not significantly different according to Tukey’s Honest Significant Differences (HSD) test (*P* ≤ 0.05)

Large lateral root length was significantly increased by drought to a similar extent in all cultivars, while small lateral root length was strongly affected by drought, cultivar and their interaction (Table 2, Fig. 8). Nahda displayed the greatest increase in small lateral root length (14-fold) in the apical segment in response to drought compared to well-watered plants (Fig. 8).

### Root Anatomy Responses to Drought

#### Nodal Roots are Thinner and Have a More Lignified Stele

**Fig. 9:**
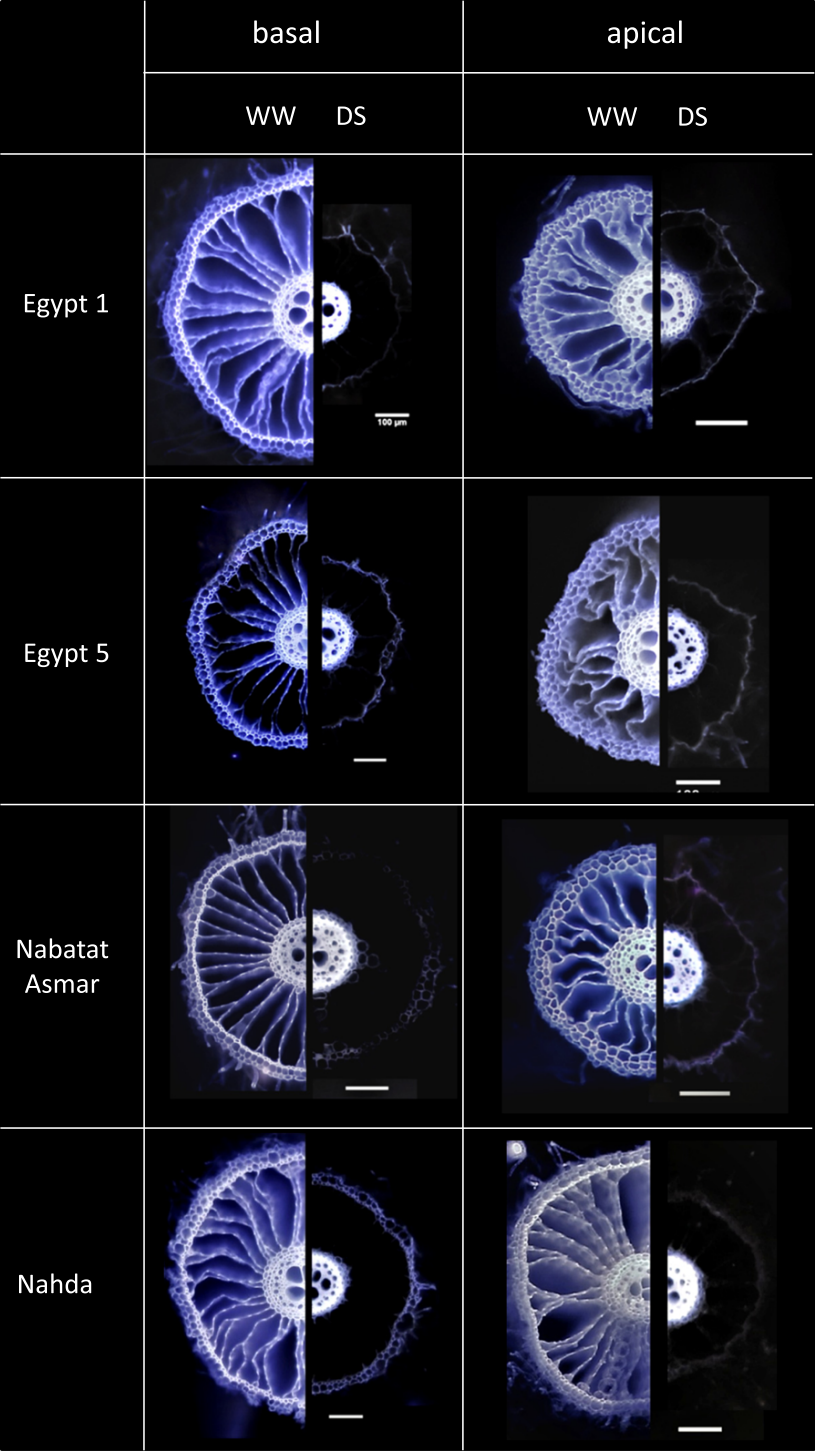
Laser Ablation Tomography (LAT) images of rice nodal root segments sampled 20 cm from the root base (basal) and 10 cm from nodal root tip (apical), under well-watered (WW) and drought stressed (DS) conditions. Each scale bar represents 100 μm

**Fig. 10:**
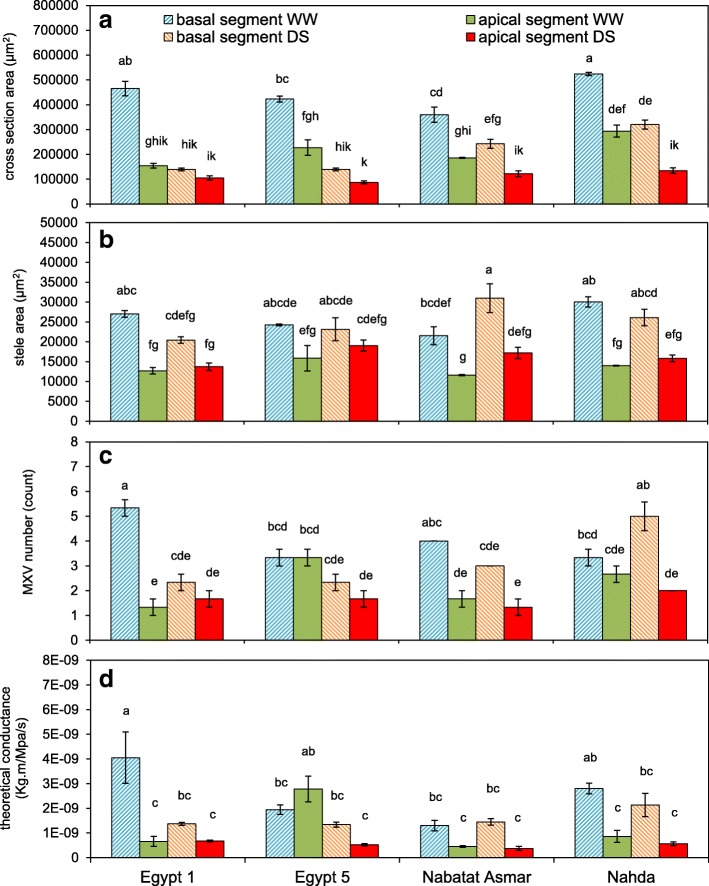
Effects of drought stress on anatomy of nodal roots of four rice cultivars grown under well-watered (WW) and drought-stressed (DS) conditions. Basal segments were sampled from 20 cm from the root base and apical segments were sampled 10 cm from the root tips. **a** cross section area; **b** stele area; **c** number of late metaxylem vessels; **d** theoretical axial conductance. Values shown are means of three replications ± SE. Means with the same letter are not significantly different according to Tukey’s Honest Significant Differences (HSD) test (*P* ≤ 0.05)

Under drought treatment, nodal root cross sectional area was 32% (Nabatat Asmar) to 71% (Egypt 1) less than that of well-watered treatment in the basal root segments, and 31.9% (Egypt 1) to 61.6% (Egypt 5) less in the apical segments (Figs. 9 and 10a). The stele area near the root apex was not significantly affected by cultivar or treatment, but there was a significant treatment x cultivar interaction for basal segment stele area (Table 2), caused by a significantly larger stele under drought in Nabatat Asmar (Fig. 10b). Stele areas were preserved at the expense of cortical areas, so that they became a much greater proportion of the total cross-sectional area (Fig. 9). The mean proportion of cross-sectional area as stele was 5.8% and 13.07% for well-watered and drought stressed basal segments, and 6.54% and 15.31% for well-watered and drought stressed apical segments, respectively.

The number of late metaxylem vessels in basal and apical nodal root segments was significantly affected by cultivar, treatment, and their interaction (Fig. 10, Table 2). Egypt 1 displayed the greatest variation between treatments and sampling positions, with fewer vessels in apical than in basal segments, and fewer vessels in basal segments under drought. Theoretical axial water conductance was reduced by 66% under drought stress in basal segments of Egypt 1, driven by the number but not the median area (Additional file 5: Figure S4) of vessels. Egypt 5 under drought, in contrast, displayed reduced conductance of the apical segments, driven by both number and median area of vessels.

**Fig. 11:**
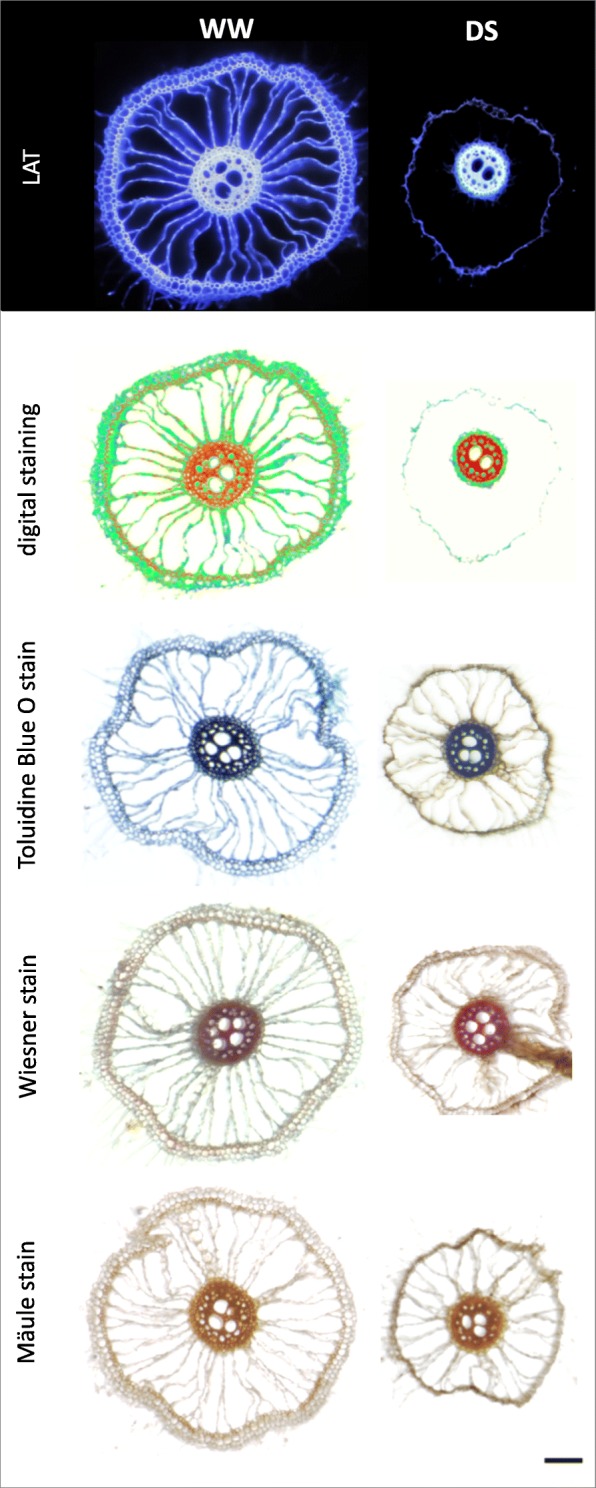
Lignin distribution pattern in response to drought stress (DS) in basal nodal root segments of cultivar Egypt 5. Variation in lignin deposition in response to DS were visualized by, from top, LAT, LAT plus digital staining, and three lignin-specific histochemical stains. Horizontal bars represent 100 μm

Drought stress led to a reduction in lignification (auto-fluorescence) of the epidermis, exodermis, and sclerenchyma in both basal and apical nodal root segments (Fig. 9). Likewise, the “spokes” of cortical cells separating aerenchyma lacunae lacked the lignification found in well-watered conditions and were no longer visible under UV illumination. We compared lignification patterns assessed by digital staining of LAT images with conventional histochemistry in nodal roots of one representative cultivar (Egypt 5) (Fig. 11, Additional file 6: Figure S5). The histochemical staining of nodal roots from dry soil by toluidine blue O, Wiesner and Mäule stains confirmed that the sclerenchyma layer and the cortical tissue between aerenchyma lacunae lacked lignin, while stele tissue accumulated more lignin than nodal roots from wet soil.

#### Anatomical Variation Along a Single Large Lateral Root

**Fig. 12:**
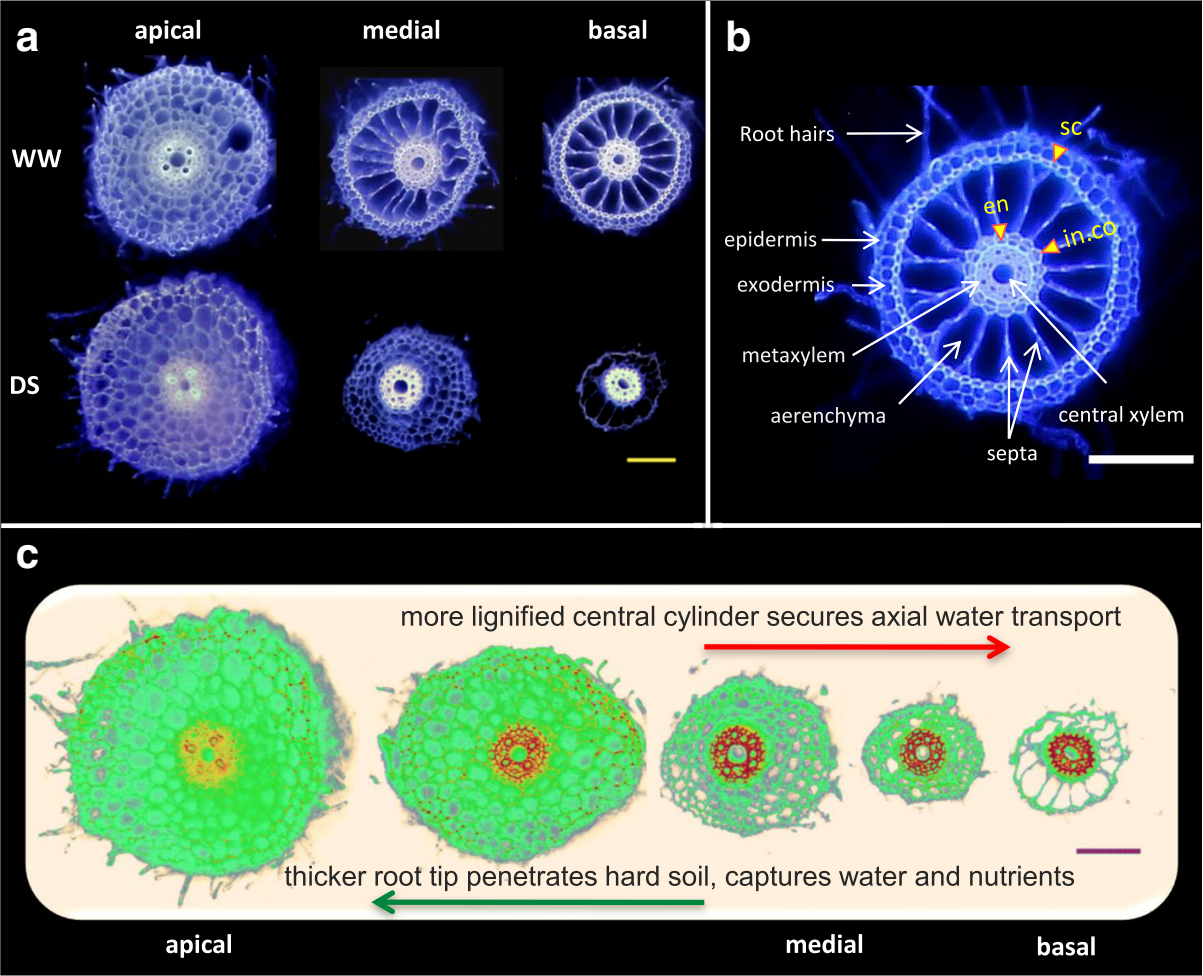
Drought stress (DS) impact on large lateral root (LLR) anatomy of Egypt 1. **a** Laser Ablation Tomography images of LLR segments sampled at three different locations: apical (2–5 mm from root tip), medial (close to the midpoint of the root), and basal (2–5 mm from point of attachment to nodal root axis). **b** anatomy of basal segment of LLR after 6 weeks in well-watered (WW) conditions showing lignification of sclerenchyma (sc), inner cortical cells (in. co), and endodermis (en). **c** model suggesting functions of observed anatomical variation using digitally stained images of Egypt 1. Red color reveals heavy lignin deposition pattern in the stele of the basal segments. Scale bars represent 100 μm

Observation of the anatomy of large lateral roots revealed increasing cross-sectional area from the root base to the apex, especially under drought (Fig. 12, Additional file 7: Figure S6, Additional file 8: Figure S7). Despite their smaller cross-sectional area (0.05–0.102 mm^2^) compared with nodal roots (0.087–0.53 mm^2^), the anatomy of large lateral roots and nodal roots was very similar (compare Figs. 9 and 12). Lignification was assessed by digital staining, which detected lignin with red color (Additional file 9: Figure S8). Like nodal roots, large lateral roots showed lignification of sclerenchyma, endodermis and inner cortical cells under well-watered conditions, while roots from drought stressed plants had less lignification outside the stele and more lignification in the stele in the older parts of the lateral root (Fig. 12, Additional file 9: Figure S8). Aerenchyma was observed in both treatments, but in drought stressed roots, it was less developed in medial segments (Fig. 12). Large lateral roots typically had a single late metaxylem vessel (Fig. 12, Additional file 8: Figure S7).

## Discussion

The Egyptian cultivars displayed a wide variation in root phenotypes when grown in large mesocosms. The accessions represent a variety of backgrounds (Additional file 1: Table S1) which might explain some of the variation in root traits. For example, varietal group Japonica cultivars are typically considered to have deeper and thicker roots than those of Indica, facilitating deep soil penetration and water acquisition in upland conditions (Gowda et al. 2011). In this study, the thickest nodal roots in this study were indeed Japonica cultivars, i.e. Yabani 47 and Nabatat Asmar. However, the two cultivars with the greatest root depth were Japonica (Nabatat Asmar) and *indica* (Egypt 5), and the shortest was Japonica (Yabani 47, Fig. 1).

Correlation among root thickness-related anatomical traits has been previously reported for rice and maize (Uga et al. 2008; Uga et al. 2009; Burton et al. 2015; Vejchasarn et al. 2016). In previous studies of an F3 population from a cross of IR64 (Indica) and Kinandang Patong (Japonica) (Uga et al. 2008) and a collection of 59 diverse accessions of cultivated rice (Uga et al. 2009), high correlations were found among stele area, total area of late metaxylem vessels, and number of metaxylem vessels. Here we confirmed correlations between root cross sectional area and stele area, and between both those traits and total metaxylem vessel area, but not with median metaxylem area or number of metaxylem vessels (Table 2). Variation related to cross-sectional area could be partially explained by varietal group differences, but clearly there is wide variation within groups as well.

There were several unexpected correlations between architectural and anatomical traits, e.g. metaxylem vessel number was positively correlated with both small and large lateral root length, but negatively correlated with maximum root depth (Table 1). In a previous study of 15 rice cultivars, metaxylem vessel number was significantly correlated with small lateral root length under low phosphorus, but not high phosphorus conditions (Vejchasarn et al. 2016). Studies with a greater number of genotypes would be required to resolve whether these correlations are typical of rice germplasm and have functional significance.

In this study, most root traits were plastic, i.e. they responded to reduced moisture availability, and in some cases the extent of the change depended on the cultivar (Table 2). Under drought, nodal root number was reduced to a similar extent in all four cultivars (Fig. 5) but nodal roots branched extensively in the deeper part of the mesocosm where more moisture was available (Figs. 6, 7 and 8). Total length per nodal root of both large and small lateral roots increased in the apical segments of nodal roots, resulting primarily from increased branching density and elongation of large lateral roots (Figs. 6, 7 and 8). Just one cultivar, Nahda, displayed increased small lateral root branching density under drought, and this occurred mostly in the basal segment exposed to drier medium (Fig. 7).

Lateral root branching in response to spatially variable water availability has been termed “hydropatterning” (Bao et al. 2014). While the term was coined to describe radially variable lateral rooting patterns when different sides of axial roots were exposed to differential moisture, plants have long been known to respond to locally available limiting resources with greater branching (Gowda et al. 2011; Rich and Watt 2013). In rice fields subjected to periodic drawdown of water levels, water is generally more available at depth. While spatial variation in lateral branching responses of deep nodal roots under stratified moisture availability has not been described in detail, others have suggested that lateral root proliferation could be important for drought tolerance in rice (Henry et al. 2011; Kano et al. 2011; Kano-Nakata et al. 2013; Tran et al. 2015). In one study of four chromosome segment substitution lines, the line that performed best under drought was the one with greatest lateral root proliferation (Kano et al. 2011). In a more recent study, root branching plasticity at different depths was investigated in 20 genotypes in the field (Sandhu et al. 2016). The authors found a positive relationship between deep root length density (driven by all root classes) and yield stability across water regimes and management strategies. Our work demonstrates that this type of plasticity can be measured in a controlled environment where it is possible to recover the entire root system, and suggests that plasticity of lateral root branching specifically in deep, higher-moisture soil could be a potential breeding objective.

Nodal roots and large lateral roots that are exposed to drought experience variation in water availability along their axes, with potential for moisture loss from the shallower portion of the root to dry soil along a water potential gradient. In this study, both the basal and apical segments of nodal roots altered their anatomical structure and composition in response to drought. Basal segments of roots exposed to drought were much thinner than those in well-watered conditions (Fig. 10). The reduction in cross-sectional area resulted from a much smaller cortex, since the stele was not significantly affected by drought (Fig. 10). Similar results were reported in anatomical evaluations of basal segments of nodal roots of Indica rice varieties exposed to well-watered or drought conditions for 30 days in two greenhouse studies (Kadam et al. 2015; Kadam et al. 2017). We also observed anatomical variation along large lateral root axes, but in this case the apical segments, which were in a wetter part of the growth medium, were similar to large lateral root segments from well-watered treatments, while the older parts of the root displayed anatomical changes similar to those of the nodal roots (Fig. 12), supporting a role for these roots in nutrient and water uptake from the deeper soil strata.

Lignification patterns in rice roots were revealed by ultraviolet (UV) illumination provided by the laser used for ablation of root tissue. UV has previously been used to visualize root anatomy based on the ability of plant cells to autofluoresce (Rebouillat et al. 2009). We adapted this method for use with laser ablation tomography, demonstrated the coincidence of UV-induced autofluorescence with conventional Maüle and phloroglucinol staining, and presented a method for digitally processing the laser ablation tomography images for enhanced visualization of lignification patterns in two species (Figs. 9, 11 and 12, Additional file 3: Figure S2 and Additional file 6: Figure S5).

Basal and apical segments of nodal roots exposed to drought displayed more lignification of the stele and less lignification of the cortex and outer layers (epidermis, exodermis, and sclerenchyma) compared with corresponding segments from well-watered plants (Fig. 9). Similar responses were found in large lateral roots, but only in the basal segments (Fig. 12). Apical segments of large lateral roots lacked an obvious sclerenchyma layer and displayed fairly uniform lignin distribution across cell types with the exception of the early metaxylem vessels. To our knowledge, there have been no previous reports of reduced lignin in outer cell layers of rice roots and increased lignification of the stele under drought. However, suberization was reported to decline in the sclerenchyma and increase in the endodermis during drought for several rice cultivars grown in the greenhouse and field (Henry et al. 2012). Suberization and lignification of the outer cell layers of rice are typically correlated (Enstone et al. 2002; Kotula et al. 2009).

The distribution and environmental responsiveness of suberized and lignified endodermal and outer cell layers suggest a role protecting root function in waterlogged and drying soils. Their importance for development of a barrier to radial oxygen loss during waterlogging is well-established (Colmer 2003). However, greater lignification and suberization of the exodermis and endodermis in stagnant solutions relative to aerated solutions were not related to hydraulic conductivity, although permeability to NaCl was significantly reduced (Garthwaite et al. 2006; Ranathunge et al. 2011). Greater thickening of sclerenchyma layers was found in irrigated compared with submerged rice roots (Mostajeran and Rahimi-Eichi 2008), which is probably related to lignification. Kondo et al. (2000) suggested that thickening of the sclerenchyma layer could play a role in structural support when the root is exposed to drying, hardening soil. In other species, e.g. sorghum and maize, the exodermis was observed to become more lignified with drought stress (Enstone et al. 2002). The fact that rice behaves differently, fortifying the endodermis and stele rather than the outer cell layers during drought, may be related to its adaptation to waterlogged conditions, including the presence of extensive aerenchyma. Fortification of the stele would maintain root function as a conduit for water and nutrient movement from lateral roots, while preventing loss of ions and water to the soil.

Basal segments of nodal roots from all four cultivars were thinner when exposed to drought, an effect that has been observed in other studies (Kadam et al. 2015; Kadam et al. 2017). The basal segments of large lateral roots were also thinner under drought, but these roots became thicker as they elongated into medium that had increasing moisture with depth (Additional file 7: Figure S6). Thinner roots may be beneficial for soil exploration because they have less nutrient and carbon cost per unit length, increasing resources available for root elongation into new soil domains (Lynch 2013), and they can explore small pores and crevices in the soil (Bengough et al. 2011). On the other hand, thicker roots have been associated with better soil penetration ability, which is important for root growth into hardened soils or through hardpans under drought conditions (Clark et al. 2008; Gowda et al. 2011; Lynch et al., 2014). In rice, these functions may depend on root class, i.e. thicker nodal roots may penetrate harder soils, while thinner lateral roots may explore fissures and pores.

Drought did not significantly affect apical stele area, and in one cultivar, Nabatat Asmar, it significantly increased basal stele area (Table 2, Fig. 10), so that the proportion of cross-sectional area as stele was significantly increased in all four cultivars. In another study, stele area was observed to increase during drought in both the greenhouse and the field (Henry et al. 2012). Conservation of stele area under drought could be beneficial for maintaining root penetration ability. In maize, stele area was positively associated with root tensile strength (Chimungu et al. 2015). In rice, maintenance of stele area and heavy fortification of the stele with lignin during drought could help roots to continue to grow as soils become harder.

The effect of drought on number of late metaxylem vessels varied among cultivars, and the median metaxylem vessel area varied in the basal segment under drought (Fig. 10, Additional file 5: Figure S4). Kadam et al. (2015) found no significant effect of drought on metaxylem diameter or number at several nodal root positions in three rice cultivars, but others have found that some rice cultivars had smaller vessels when exposed to drought (Yambao et al. 1992; Mostajeran and Rahimi-Eichi 2008; Henry et al. 2012). Smaller vessels would be expected protect the xylem from cavitation, moderate water movement to the shoot, and help maintain a moist rhizosphere for continued root growth and water and nutrient uptake. Plasticity of vessel size could be useful to provide these advantages under drought while permitting adequate water transport to the shoot to support rapid growth under well-watered conditions.

Cultivated rice displays wide variation for root architectural and anatomical traits, as well as variation for plasticity of these traits. We propose that under moderate progressive drought and under reduced water-usage management strategies, where water potential in the upper soil layers declines but more moisture is available at depth, rice crops will benefit from deep soil exploration via extension and branching of large lateral roots, while minimizing development of additional nodal roots. Patterns of lignification and suberization would additionally help to maintain water uptake from deep soil horizons while minimizing losses to dry soil in the shallower layers. These traits require further examination to confirm their utility in agricultural fields.

## Additional files

Additional file 1: **Table S1.** Egyptian cultivars used in the study, with accession numbers and flowering times from the U.S. National Plant Germplasm System, and subpopulation assignments. (DOCX 21 kb)

Additional file 2: **Figure S1.** Xylem anatomy of 11 Egyptian rice cultivars grown in the greenhouse under well-watered conditions: **a** median metaxylem vessel area and **b** metaxylem vessel number. Values shown are means of three replications ± SE. Means with the same letter are not significantly different according to Tukey’s Honest Significant Differences (HSD) test (*P* ≤ 0.05). (PPTX 53 kb)

Additional file 3: **Figure S2. a** Laser Ablation Tomography (LAT) image of a sweet corn nodal root segment sampled 15 cm from the root base, and **b** the same image digitally stained. Lignin deposition is indicated as red colored areas. Scale bar represents 100 μm. (PPTX 1.29 mb)

Additional file 4: **Figure S3.** Schematic representation of mesocosms used for rice growth and imposition of drought. Volumetric water content (θ*v*) of the medium in the upper and lower parts of the mesocosms are indicated. These measurements were recorded using TDR probes after 6 weeks growth, including the final 4 weeks without additional water. Probes were inserted 25 cm from the surface under well-watered conditions, and at 25 cm and 100 cm depth for drought stress treatments. Values shown are ranges of three replications. (PPTX 257 kb)

Additional file 5: **Figure S4.** Effects of drought stress on median metaxylem vessel area in basal segments of nodal roots of four rice cultivars grown under drought-stressed (DS) conditions. Values shown are means of three replications ± SE. Means with the same letter are not significantly different according to Tukey’s Honest Significant Differences (HSD) test (*P* ≤ 0.05). There were no significant differences in median metaxylem vessel areas in apical segments of DS plants or in any segments of well-watered plants. (PPTX 42 kb)

Additional file 6: **Figure S5.** Nodal root cross sections taken from basal segments and stained with Weisner stain show variation in lignin deposition in the stele and endodermis between well-watered (WW) and drought stress (DS) conditions. Black arrowheads indicate the endodermis. (PPTX 1.67 mb)

Additional file 7: **Figure S6.** Cross sectional areas of large lateral roots of cultivar Egypt 1 at distal, medial and basal positions. Values shown are means of three replications ± SE. Means with the same letter are not significantly different according to Tukey’s Honest Significant Differences (HSD) test (*P* ≤ 0.05). (PPTX 42 kb)

Additional file 8: **Figure S7.** LAT (**a**) and digitally stained (**b**) images of a basal segment cross-section of a large lateral root under well-watered conditions showing lignification of sclerenchyma (sc), inner cortical area (in.co) and endodermis (en). Horizontal bars represent 100 μm. (PPTX 599 kb)

Additional file 9: **Figure S8. a** Portion of scanned root system of Egypt 1 grown under drought stress with large lateral root (LLR) anatomical sampling positions indicated. **b** LAT images from a single LLR sampled from apical (1) to basal (5) positions. Scale bar in **b** represents 100 μm. (PPTX 535 kb)

## Abbreviations

CPD: Critical point dryer
DS: Drought-stressed
HSD: Honest significant difference
LAT: Laser ablation tomography
LLR: Large lateral root
LRBD: Lateral root branching density
SLR: Small lateral root
TDR: Time domain reflectometry
UV: Ultraviolet
WW: Well-watered
