# Supplementary material for: Progressive drought alters architectural and anatomical traits of rice roots
Source: Rice (N Y). 2018 Dec 4;11:62. doi: 10.1186/s12284-018-0252-z (PMC6277260; doi:10.1186/s12284-018-0252-z)
Supplement: Supplementary file 1 — Table S1. Egyptian cultivars used in the study, with accession numbers and flowering times from the U.S. National Plant Germplasm System, and subpopulation assignments. (DOCX 21 kb) [file 12284_2018_252_MOESM1_ESM.docx]

Table S1. Egyptian cultivars used in the study, with accession numbers and flowering times from the U.S. National Plant Germplasm System, and subpopulation assignments.

| Cultivar | Accession ID | Days to Flower | Subpopulation assignment | Reference | |
| --- | --- | --- | --- | --- | --- |
| Giza 14 | PI 439130 | 133 | *aus* | (Agrama et al. 2010) | |
| Giza 159 | GSOR 311316 | 130 | TEJ | (Agrama et al. 2010) | |
| Yabani Montakhab 7 (mon. 7) | GSOR 301207 | 109 | Admix† | (Zhao et al. 2010) | |
| Giza 180 (Sakha 1) | PI 439135 | 116 | Indica | (Dalrymple 1986; Anonymous 1987) | |
| Yabani 47 | PI 233881 | 124 | Japonica | (Anonymous 1987)* | |
| Arabi | PI 439121 | 128 | Admix TRJ TEJ | (Agrama et al. 2010; Zhao et al. 2010) | |
| Egypt 6 | PI 431163 | 104 | Japonica | Dr. Abd Allah Abd Elnaby, ARC, Egypt, personal commun. | |
| Nabatat Asmar | PI 439139 | 93 | TRJ | (Agrama et al. 2010) |  |
| Nahda | PI 329136 | 124 | TEJ | (Agrama et al. 2010) | |
| Egypt 5 | PI 431162 | 118 | *indica* | (Agrama et al. 2010) | |
| Egypt 1 | PI 431158 | 92 | *indica* | (Agrama et al. 2010) | |

Abbreviations and font designations: TEJ: temperate japonica; TRJ: tropical japonica; upper case Indica: varietal group indica; lower case *indica*: subpopulation *indica*.

*Varietal group inferred based on parentage, amylose content, and grain length, and Arabic translation of Yabani as "Japanese".

†Listed as TEJ by (Agrama et al. 2010)

**References cited for supplemental table 1**

Agrama HA, Yan W, Jia M, et al. (2010) Genetic structure associated with diversity and geographic distribution in the USDA rice world collection. Nat Sci 2:247.

Anonymous (1987) Rice Farming Systems. International Rice Research Institute

Dalrymple DG (1986) Development and spread of high-yielding rice varieties in developing countries. Int. Rice Res. Inst.

Zhao K, Wright M, Kimball J, et al. (2010) Genomic Diversity and Introgression in O. sativa Reveal the Impact of Domestication and Breeding on the Rice Genome. PLoS One 5:e10780.
