# Supplementary material for: Progressive drought alters architectural and anatomical traits of rice roots
Source: Rice (N Y). 2018 Dec 4;11:62. doi: 10.1186/s12284-018-0252-z (PMC6277260; doi:10.1186/s12284-018-0252-z)
Supplement: Supplementary file 2 — Figure S1. Xylem anatomy of 11 Egyptian rice cultivars grown in the greenhouse under well-watered conditions: a median metaxylem vessel area and b metaxylem vessel number. Values shown are means of three replications ± SE. Means with the same letter are not significantly different according to Tukey’s Honest Significant Differences (HSD) test (P ≤ 0.05). (PPTX 53 kb) [file 12284_2018_252_MOESM2_ESM.pptx]

## Slide 1
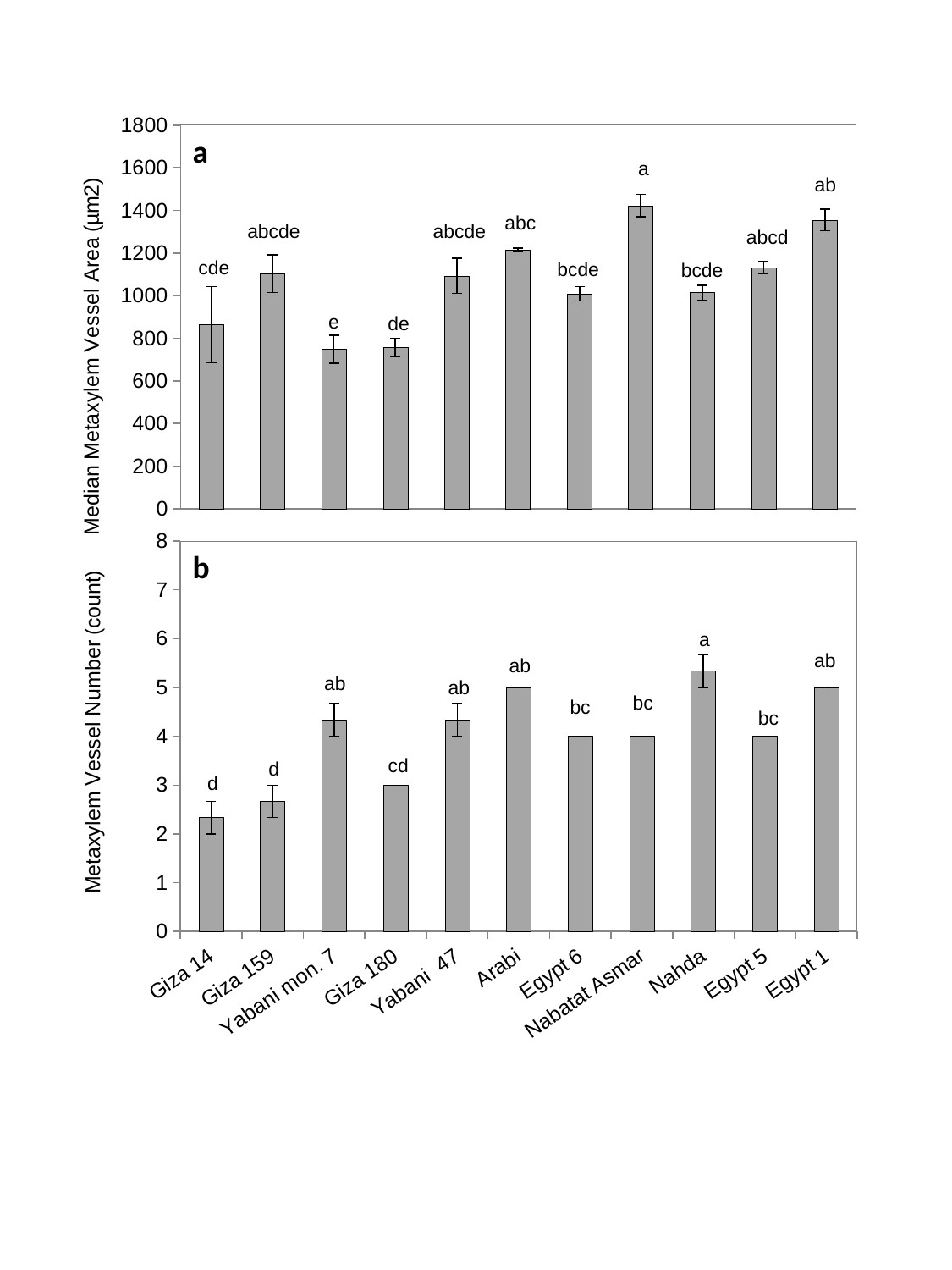

### Chart
| Category | |
|---|---|
| Giza 14 | 864.459352777778 |
| Giza 159 | 1103.187292333324 |
| Yabani mon. 7 | 748.777005 |
| Giza 180 | 757.4035515555555 |
| Yabani mon. 47 | 1092.7812953666664 |
| Arabi | 1214.1124067333244 |
| Egypt 6 | 1008.764509 |
| Nabata Asmar | 1422.6763098333263 |
| Nahda | 1013.8610292111081 |
| Egypt 5 | 1131.3950162499998 |
| Egypt 1 | 1355.1835104666668 |a
ab
abc
abcde
abcde
abcd
cde
bcde
bcde
e
de
a
### Chart
| Category | |
|---|---|
| Giza 14 | 2.3333333333333335 |
| Giza 159 | 2.6666666666666665 |
| Yabani mon. 7 | 4.33333333333335 |
| Giza 180 | 3.0 |
| Yabani 47 | 4.33333333333335 |
| Arabi | 5.0 |
| Egypt 6 | 4.0 |
| Nabatat Asmar | 4.0 |
| Nahda | 5.33333333333335 |
| Egypt 5 | 4.0 |
| Egypt 1 | 5.0 |a
ab
ab
ab
ab
bc
bc
bc
cd
d
d
b
