# Supplementary figures and images for: Progressive drought alters architectural and anatomical traits of rice roots
Source: Rice (N Y). 2018 Dec 4;11:62. doi: 10.1186/s12284-018-0252-z (PMC6277260; doi:10.1186/s12284-018-0252-z)

## Slide 1
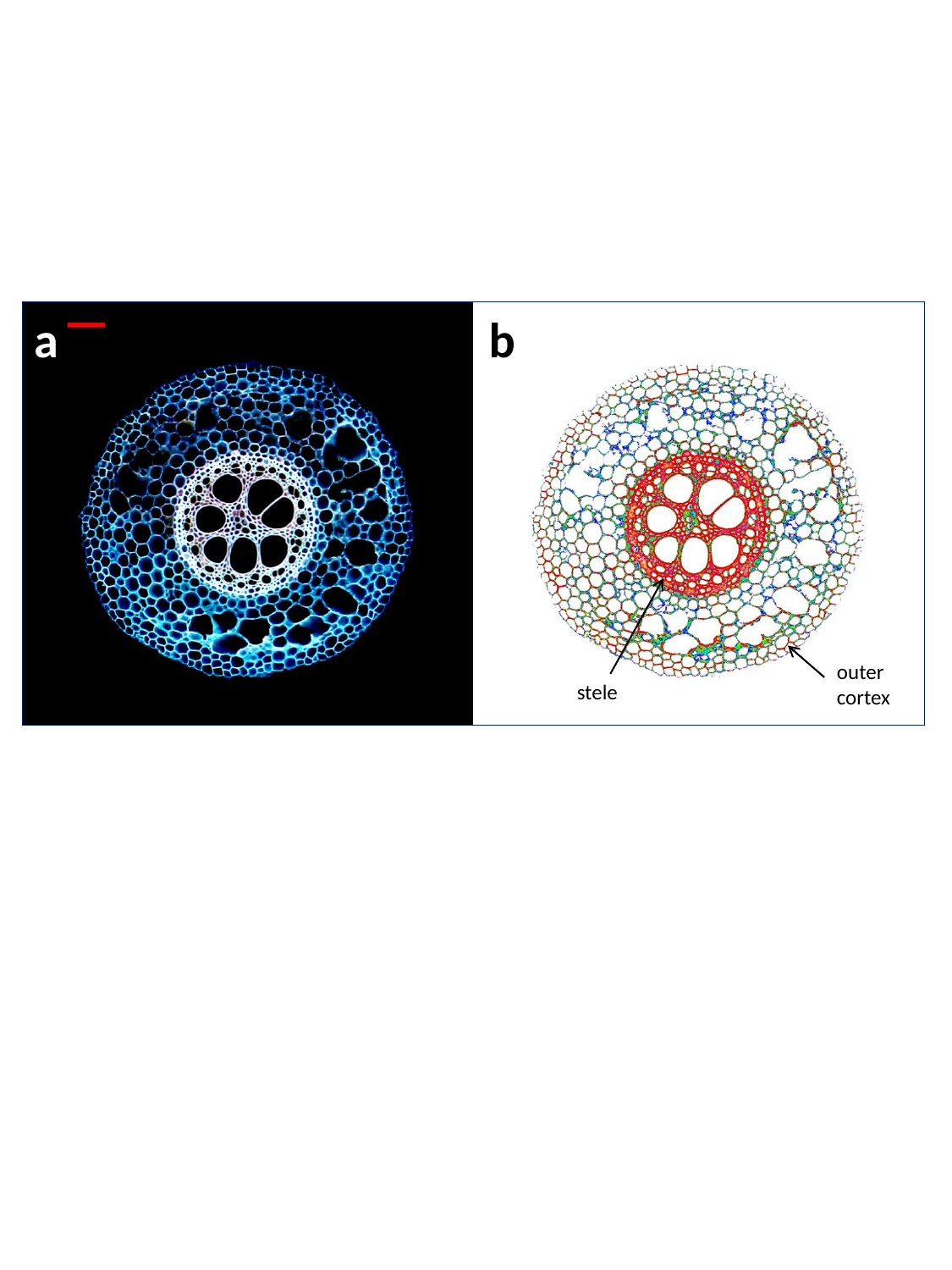

a
b
outer cortex
stele

Supplement: Supplementary file 3 — Figure S2. a Laser Ablation Tomography (LAT) image of a sweet corn nodal root segment sampled 15 cm from the root base, and b the same image digitally stained. Lignin deposition is indicated as red colored areas. Scale bar represents 100 μm. (PPTX 1.29 mb) [file 12284_2018_252_MOESM3_ESM.pptx]

## Slide 1
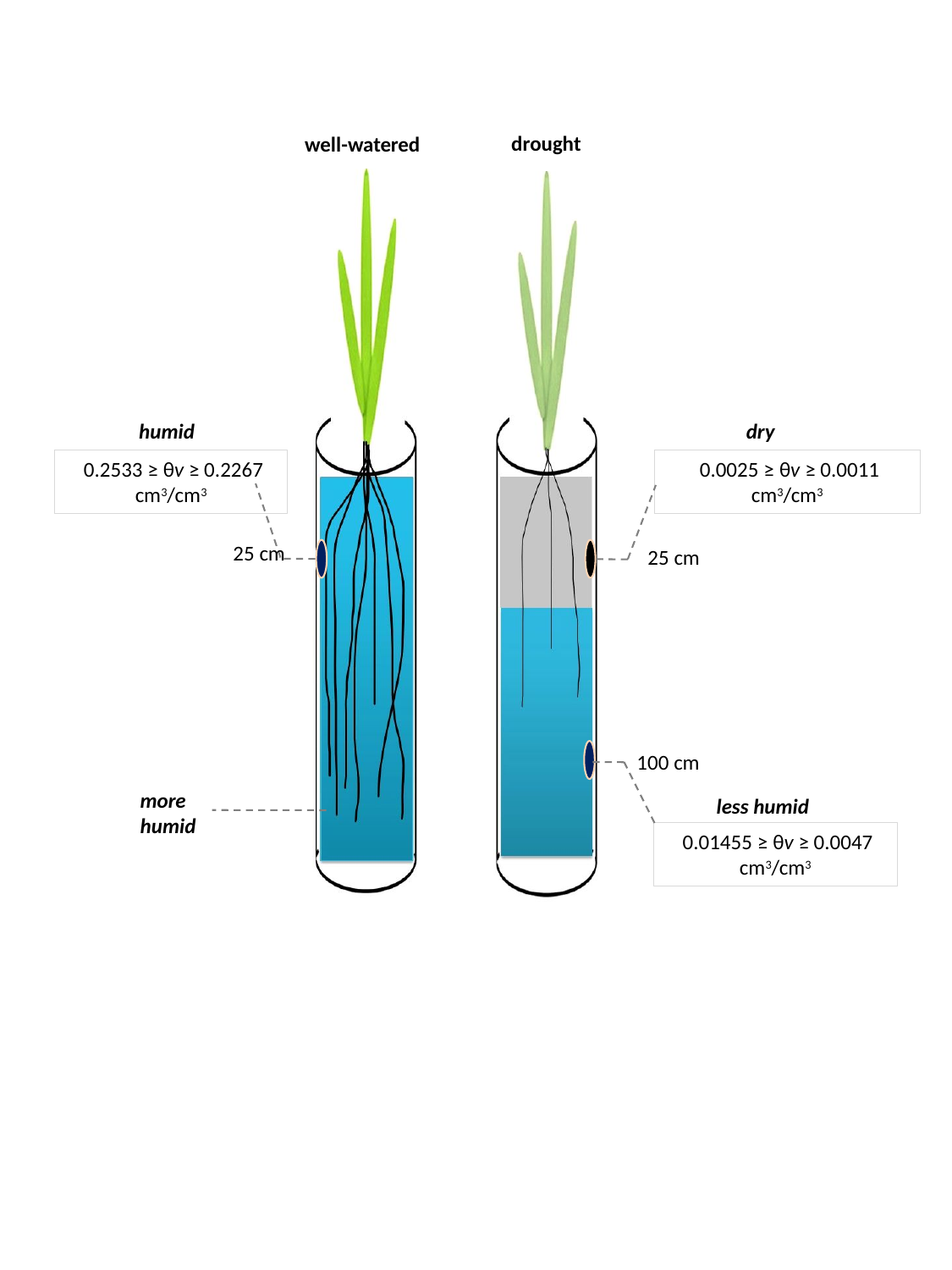

drought
well-watered
humid
dry
 0.2533 ≥ θv ≥ 0.2267 cm3/cm3
 0.0025 ≥ θv ≥ 0.0011 cm3/cm3
25 cm
25 cm
100 cm
more humid
less humid
 0.01455 ≥ θv ≥ 0.0047 cm3/cm3

Supplement: Supplementary file 4 — Figure S3. Schematic representation of mesocosms used for rice growth and imposition of drought. Volumetric water content (θv) of the medium in the upper and lower parts of the mesocosms are indicated. These measurements were recorded using TDR probes after 6 weeks growth, including the final 4 weeks without additional water. Probes were inserted 25 cm from the surface under well-watered conditions, and at 25 cm and 100 cm depth for drought stress treatments. Values shown are ranges of three replications. (PPTX 257 kb) [file 12284_2018_252_MOESM4_ESM.pptx]

## Slide 1
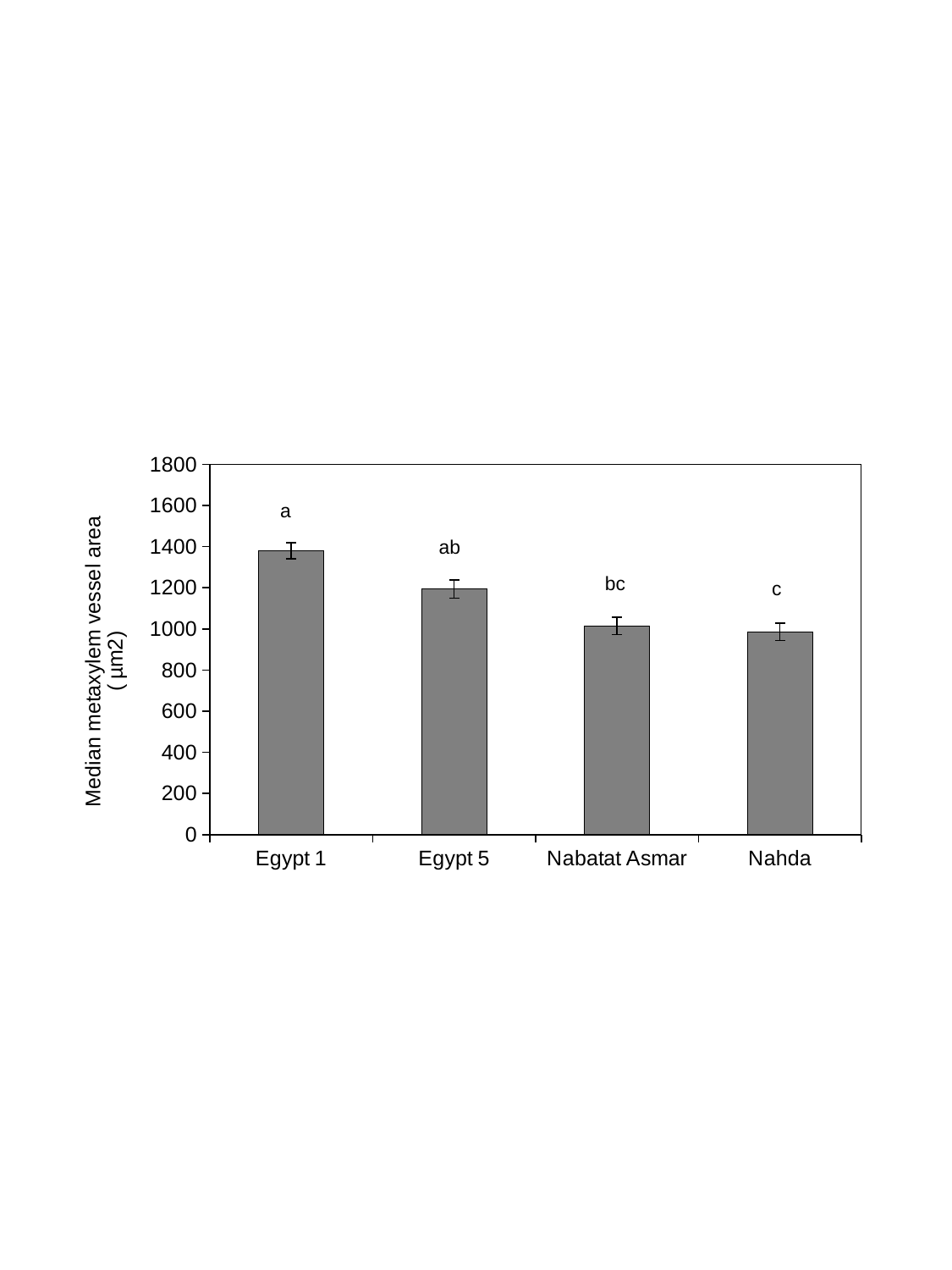

### Chart
| Category | |
|---|---|
| Egypt 1 | 1380.7401095000002 |
| Egypt 5 | 1194.8072111666681 |
| Nabatat Asmar | 1015.1573553333334 |
| Nahda | 986.0267528333334 |a
ab
bc
c

Supplement: Supplementary file 5 — Figure S4. Effects of drought stress on median metaxylem vessel area in basal segments of nodal roots of four rice cultivars grown under drought-stressed (DS) conditions. Values shown are means of three replications ± SE. Means with the same letter are not significantly different according to Tukey’s Honest Significant Differences (HSD) test (P ≤ 0.05). There were no significant differences in median metaxylem vessel areas in apical segments of DS plants or in any segments of well-watered plants. (PPTX 42 kb) [file 12284_2018_252_MOESM5_ESM.pptx]

## Slide 1
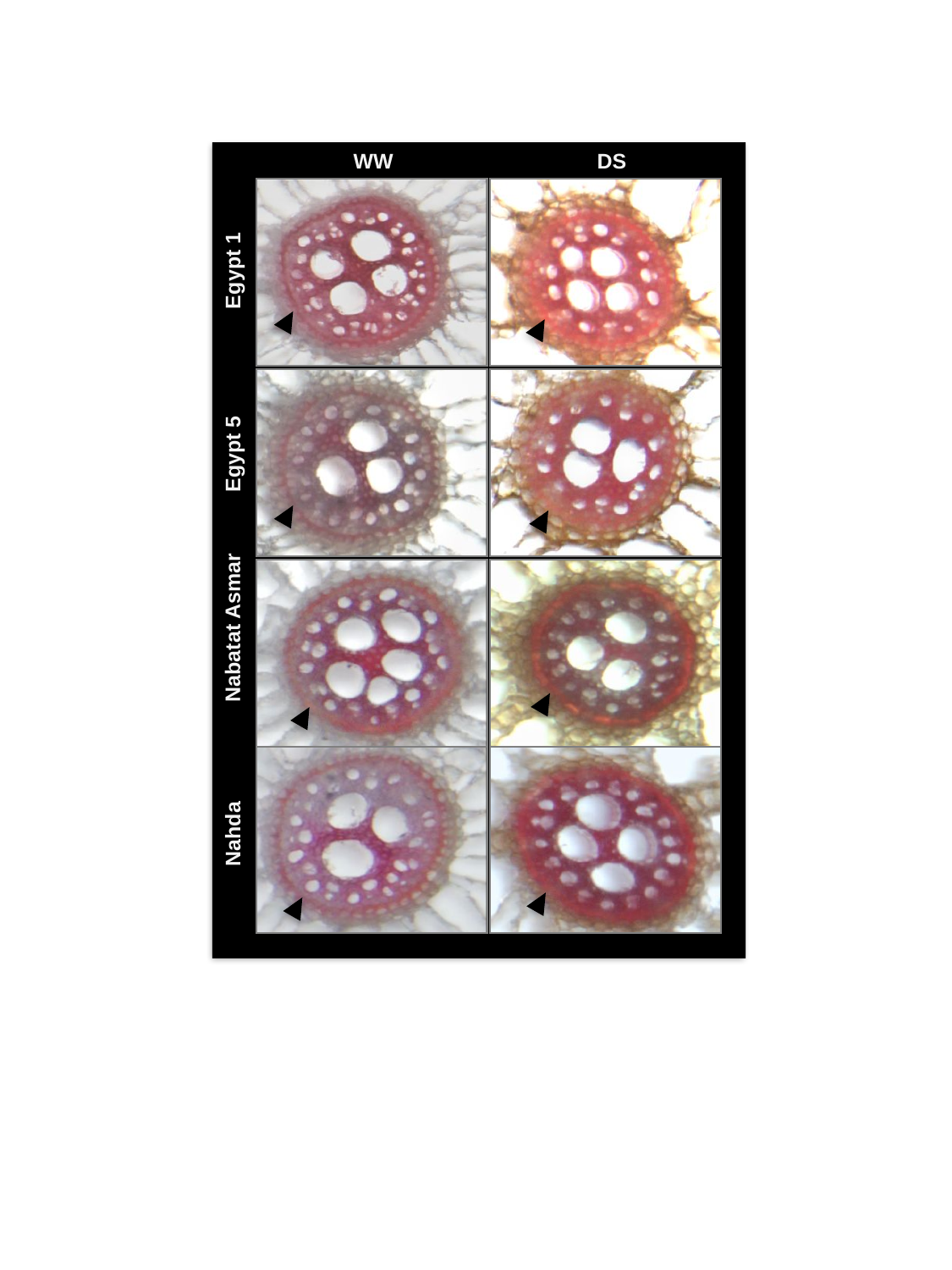

WW
DS
Egypt 1
Egypt 5
Nabatat Asmar
Nahda

Supplement: Supplementary file 6 — Figure S5. Nodal root cross sections taken from basal segments and stained with Weisner stain show variation in lignin deposition in the stele and endodermis between well-watered (WW) and drought stress (DS) conditions. Black arrowheads indicate the endodermis. (PPTX 1.67 mb) [file 12284_2018_252_MOESM6_ESM.pptx]

## Slide 1
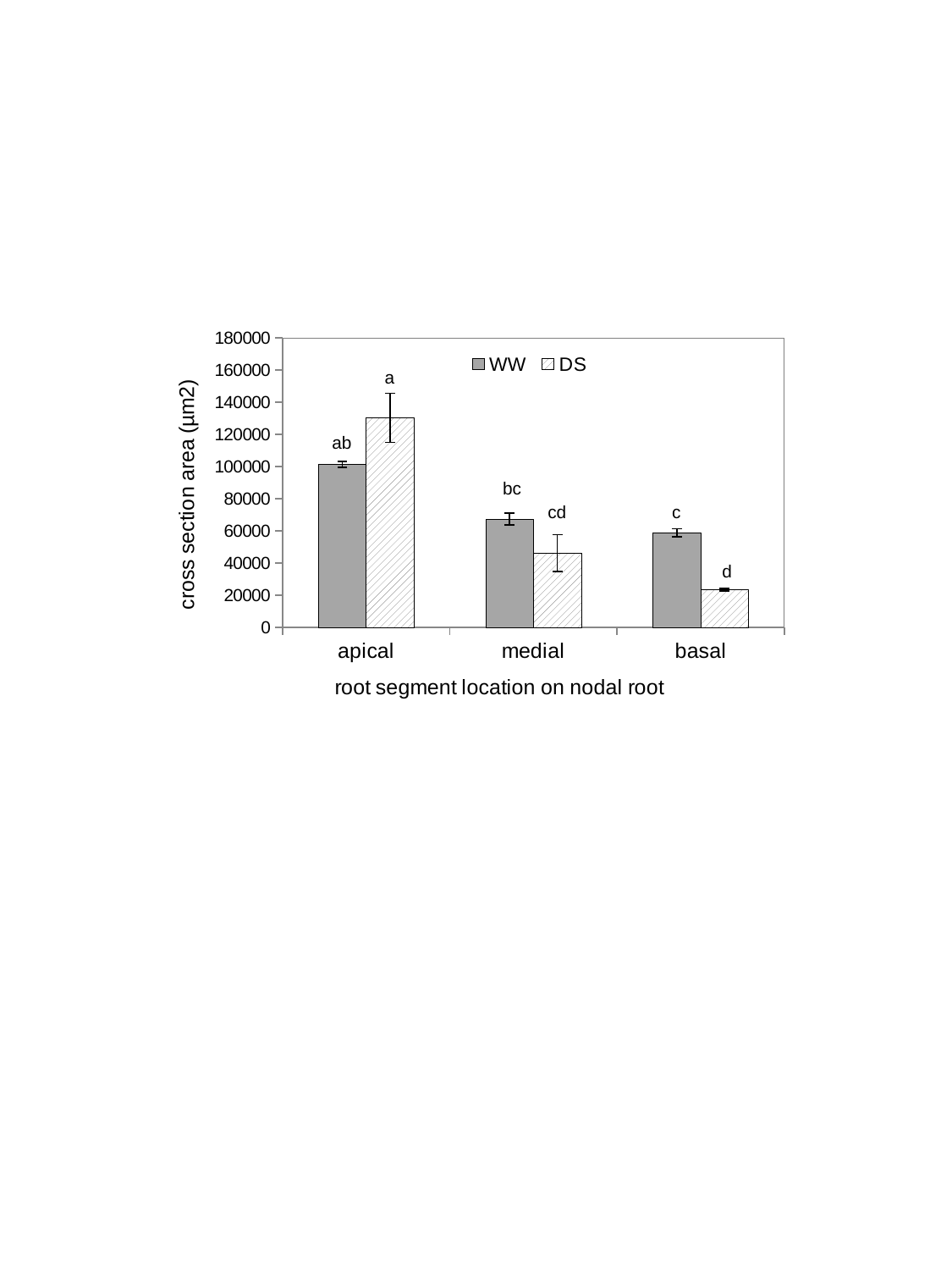

### Chart
| Category | WW | DS |
|---|---|---|
| apical | 101273.121583 | 130394.19867266667 |
| medial | 67441.893751 | 46227.62562366646 |
| basal | 58881.169874666586 | 23430.214344666663 |a
ab
bc
cd
c
d

Supplement: Supplementary file 7 — Figure S6. Cross sectional areas of large lateral roots of cultivar Egypt 1 at distal, medial and basal positions. Values shown are means of three replications ± SE. Means with the same letter are not significantly different according to Tukey’s Honest Significant Differences (HSD) test (P ≤ 0.05). (PPTX 42 kb) [file 12284_2018_252_MOESM7_ESM.pptx]

## Slide 1
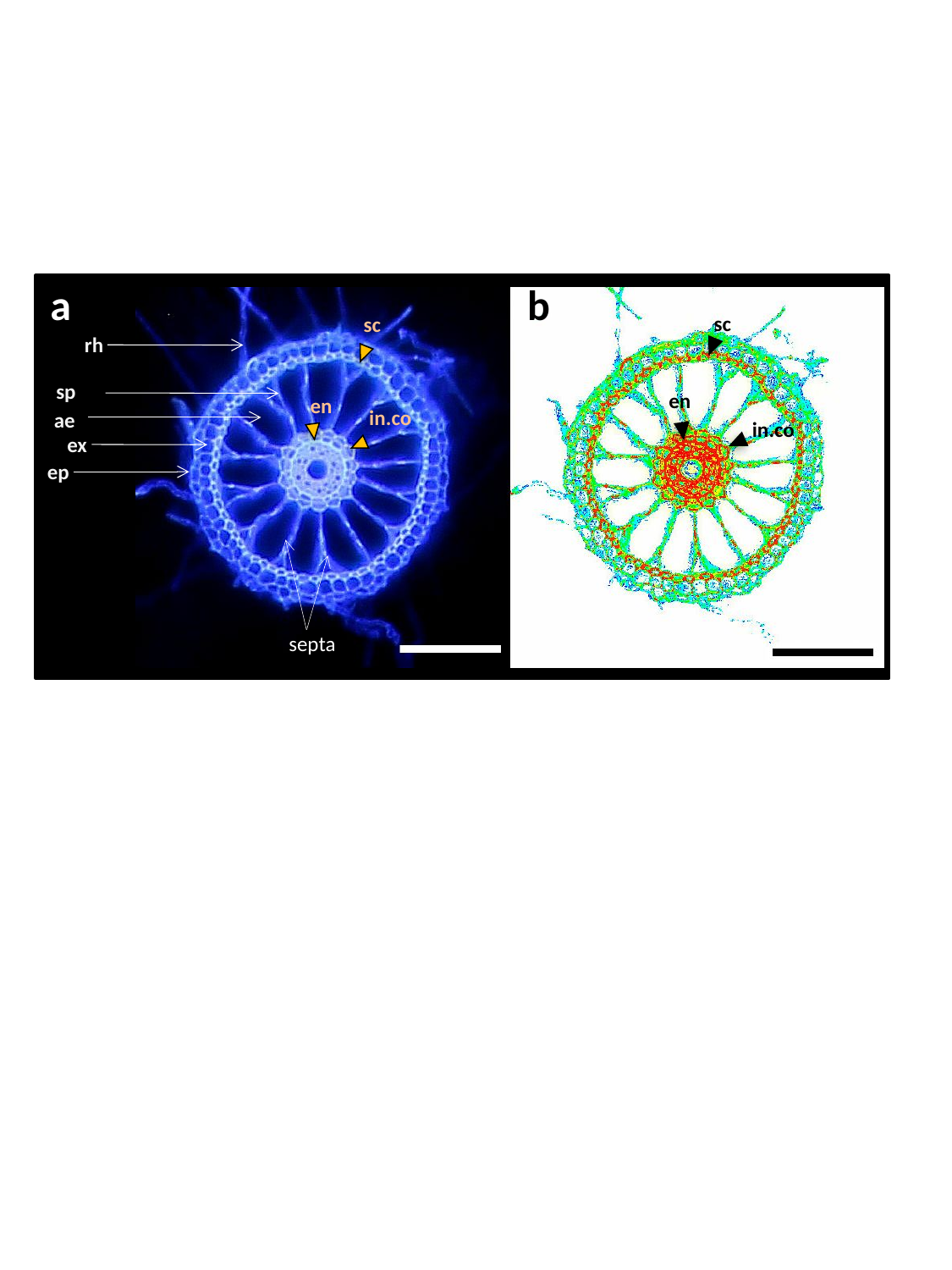

a
b
sc
sc
rh
sp
en
en
in.co
ae
in.co
ex
ep
septa

Supplement: Supplementary file 8 — Figure S7. LAT (a) and digitally stained (b) images of a basal segment cross-section of a large lateral root under well-watered conditions showing lignification of sclerenchyma (sc), inner cortical area (in.co) and endodermis (en). Horizontal bars represent 100 μm. (PPTX 599 kb) [file 12284_2018_252_MOESM8_ESM.pptx]

## Slide 1
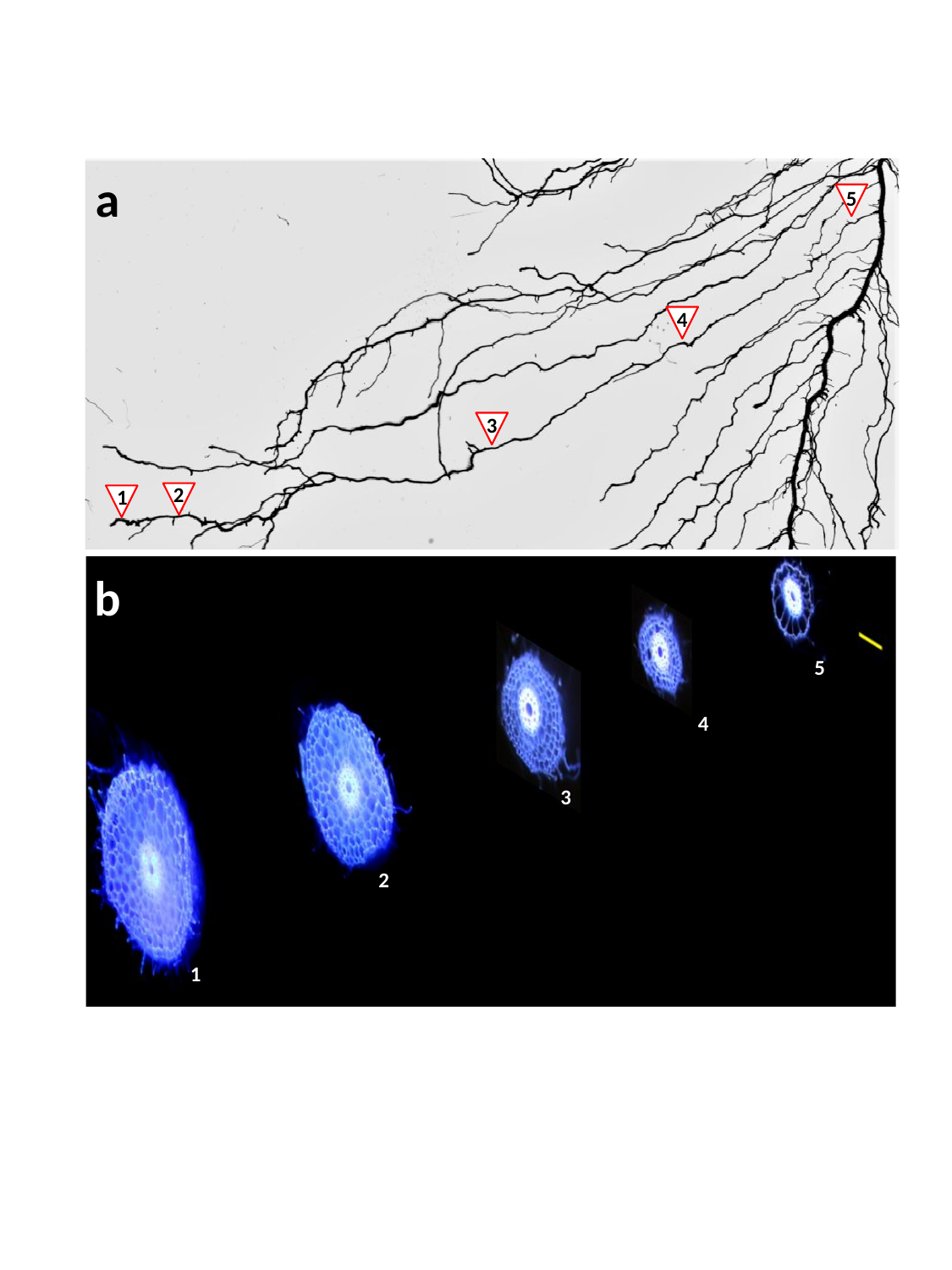

a
b
5
4
3
2
1
5
4
3
2
1

Supplement: Supplementary file 9 — Figure S8. a Portion of scanned root system of Egypt 1 grown under drought stress with large lateral root (LLR) anatomical sampling positions indicated. b LAT images from a single LLR sampled from apical (1) to basal (5) positions. Scale bar in b represents 100 μm. (PPTX 535 kb) [file 12284_2018_252_MOESM9_ESM.pptx]
